# Effects of Pressurized Water Aging on Reciprocating Friction and Wear of FDM 3D-Printed PLA and Glass Fiber Reinforced PLA Composites

**DOI:** 10.3390/polym18030406

**Published:** 2026-02-04

**Authors:** Sinan Fidan, Satılmış Ürgün, Nevin Gamze Karsli, Taner Yilmaz, Mustafa Özgür Bora, Mehmet İskender Özsoy

**Affiliations:** 1 Department of Airframe and Powerplant Maintenance, Faculty of Aeronautics and Astronautics, Kocaeli University, Kocaeli 41001, Türkiye; sfidan@kocaeli.edu.tr (S.F.); ozgur.bora@kocaeli.edu.tr (M.Ö.B.); 2 Department of Aviation Electrics and Electronics, Faculty of Aeronautics and Astronautics, Kocaeli University, Kocaeli 41001, Türkiye; urgun@kocaeli.edu.tr; 3 Department of Aerospace Engineering, Faculty of Aeronautics and Astronautics, Kocaeli University, Kocaeli 41001, Türkiye; gamze.karsli@kocaeli.edu.tr; 4 Department of Mechanical Engineering, Engineering Faculty, Kocaeli University, Kocaeli 41001, Türkiye; taner.yilmaz@kocaeli.edu.tr; 5 Department of Mechanical Engineering, Faculty of Engineering, Sakarya University, Sakarya 54050, Türkiye

**Keywords:** fused deposition modeling (FDM), glass-fiber-reinforced PLA, pressurized water aging, reciprocating friction and wear, water absorption

## Abstract

This study evaluates 10 bar water aging effects on reciprocating tribology of FDM-printed PLA and PLA with 10 and 15 wt.% glass fiber (GF). Water uptake was Fickian, and saturation mass rose from 0.0845 g (PLA) to 0.1625 g and 0.295 g (10 and 15 wt.% GF). Reciprocating tests at 40 N over 100 m at 0.5 and 1 Hz showed immersion time drives coefficient of friction (COF) and wear. At 0.5 Hz, neat PLA stabilized at COF 0.65 to 0.70 but increased to about 0.75 to 0.80 after 7-day; PLA + 10 wt.% GF reached about 0.80 to 0.82 after 14-day to 28-day. GF reduced unaged wear depth from about 125 µm to about 85 to 96 µm, yet 28-day aging increased depths to about 129 to 132 µm for both GF levels at 0.5 Hz. At 1 Hz, neat PLA peaked at about 235 to 240 µm depth after 7-day, whereas 15 wt.% GF reached about 160 µm after 28-day. Factorial analysis showed that wear scar width was primarily influenced by immersion time, accounting for 76.02% of the variation in the data, clearly evidencing strong dependence on the environment. Scanning electron microscopy (SEM), differential scanning calorimetry (DSC), glass transition temperature (Tg), and the melting temperature (Tm) support the occurrence of a transition from volume to interface-dominated damage with aging, while Tg and Tm remain unaffected.

## 1. Introduction

Additive manufacturing (AM) techniques, especially the process popularly known as fused deposition modeling (FDM) due to its cost-effectiveness and flexibility, are gaining popularity for the manufacturing of functional parts [1,2,3,4]. Properties of FDM samples have been found to be critically dominated by both the material properties and process characteristics like interlayer adhesions or porosity [5,6]. Hence, understanding the relationship between the microstructural development and related mechanical properties has become vital for optimizing printing parameters with the guarantee of producing serviceable mechanical parts [7,8,9,10]. Although engineering plastics such as polyamide 6 (PA 6), polylactic acid (PLA) and acrylonitrile butadiene styrene (ABS), which are the most commonly used materials in 3D printing [11,12,13,14,15], PLA, which is a biodegradable polyester, is found to have excellent mechanical properties like tensile strength/elastic modulus but with low ductility [16,17]. In addition, addition of additives/plasticizers or fillers becomes an essential parameter by improving functionality [18,19]. Bohun et al. [20] investigated the tribological reinforcement of polylactic acid (PLA) composites in additive manufacturing using a clay filler. The paper discusses the impact of varying amounts of additive and the resultant infill patterns on 3D printing on wear resistance and the friction coefficient under dry sliding. The study concludes that a mixture with 0.1 g of additive per 50 g of PLA with a Grid 90/85 infill pattern leads to a satisfactory trade-off between low wear rate and weight. The research study led by Santo et al. [21] studied the tribological properties of PLA 3D print composites with added graphene using electrochemical (EG) and chemical (HG) exfoliation methods. The study concludes that a 0.5 weight% filler amount reduces the friction coefficient by 76%. A shift from abrasion to adhesive wear was also reported when PLA was converted to its composite form. Filler type has a significant impact on wear rate; however, the solvent had a minimal effect. In the study conducted by Sánchez-Rodríguez et al. from reference [22], the use of a solid ionic liquid, 1-butyl-1-methylpyrrolidinium hexafluorophosphate, as an additive in the fabrication of a polylactic acid (PLA) specimen fabricated through the FDM process has been explored. The use of the ionic liquid improved. Additionally, the addition improved the interlayer bonding properties. The study concluded that a maximum average reduction of 45% in friction was experienced when sliding perpendicular to the additive path. The ionic liquid also improved the distance to reach a stationary friction state. The study showed that a superficial polishing effect with minimal wear was experienced on the printed specimens. Rashia et al. [23] investigate the mechanics of glass-fiber-reinforced PLA specimens 3D printed with FDM. The study analyzed the effect of varying infill density (40%, 50%, 60%) and angle of raster path (0°, 45°, 90°) on mechanical properties. Mechanical behavior of specimens was modified with a 60% infill density of 0° and 90° angles. The lowest rate of wear was experienced when the raster path was at a 0° angle. The study concluded that FDM specimens possess poor mechanical properties when contrasted to traditionally manufactured specimens. This was addressed in the study when glass fiber reinforcement was used to improve mechanical behavior and tribological properties.

An in situ production method for glass-fiber-reinforced polylactic acid (PLA-GF) composites based on fused deposition modeling (FDM) is discussed by Liow et al. [24]. The particular study focuses on the analysis of the friction and wear characteristics of PLA-GF composites at different linear velocities and raster angles to the sliding direction, using the pin-on-disk test rig. The increasing usage of thermoplastic composites in tribology can be largely credited to the property of self-lubrication, along with the simplicity of fabrication. The paper by Suresh et al. [25] investigates the dry sliding wear characteristics of neat polylactic acid and short-carbon fiber-reinforced polylactic acid composites prepared by FDM. The wear resistance of the composites prepared by FDM is typically evaluated using the pin-on-disc machine, and studies are performed as a function of load, sliding speed, and sliding distance using response surface methodology. The study shows a 70% decrease in wear of the composites, owing to the fiber reinforcements, which exhibit grooves, fiber retraction, or slight pull-out. Ichakpa et al. [26] examine the application of 3D printing of PA6 composites, incorporating glass fibers (GF) and graphene oxide (GO), for electric vehicle battery case fabrication. The paper focuses particularly on augmenting the interfacial strength and interlaminar strength at different environmental conditions, like ambient, wet, and higher temperature. The findings disclose that the addition of the highest strength, compared to other environmental treatments, is obtained by glass fibers, and the adding of 0.1% of the graphene oxide component remarkably increases the tensile strength of the composite in all environmental situations, making the PA6 composite. Specifically, it is appropriate for exposure to water.

Composite tribology studies assume prominence in the longer-term usage of the components in the aerospace, automotive, and marine industry by studying the wear process under varied operating conditions which forms a major part of tribology and helps in the selection of appropriate materials [27,28]. The wear resistance of polymers also depends on the mechanical property, operating and environmental factors, friction counter-face materials, and surface roughness [29,30]. In addition, presence of ultraviolet, water, and mechanical loading can accelerate the degradation process of the polymer through the aging process [31]. The absorption of water in the case of polymers takes place in the amorphous phase and affects the chemical, mechanical, and physical properties accordingly [32]. Kakanuru and Pochiraju [33] analyzed the degradation of FDM-printed polylactic acid, silicon carbide and polylactic acid (SiC/PLA) composites, and acrylonitrile butadiene styrene (ABS) composites under hydro-thermal aging at 50 °C and 70 °C, respectively. The study implies that pure polylactic acid exhibits large degradation, and increased silicon carbide content increases the thermostability, thereby reducing the water diffusivity. On the contrary, high silicon carbide content may decrease the mechanical properties. The ABS exhibits less tensile strength but increases the moisture content, thereby reducing the stiffness. Fragassa et al. [34] offer a compilation of the latest research findings regarding the mechanisms of friction, wear, and degradation in bio composites, developed for use in the marine field. Indeed, despite the preference shown to the sustainability offered by this kind of material, environmental conditions in the marine field tend to limit their performance. The paper discusses the durability offered by this kind of material and investigates the progress reached regarding surface treatments, formulating techniques, and the improvement of resistance. Ainin et al. [35] have investigated the effect of water uptake on the mechanics of 3D-printed sandwich composites, consisting of PLA, PLA-C, and PLA-W. The findings confirm that the highest level of absorption is shown by the hydrophilic material PLA-W, hence the lowest values of the compressive strength and modulus. It has been found that the energy absorption capacity and controlled deformation of PLA-C were the best. The resistance of PLA-based bio composites against hygrothermal exposure has come into the spotlight, especially when the material operates in hot and damp climates. There are indications that the absorption of water can affect the inter binding properties of jute or flax-reinforced composites based upon PLA [36].

Although the literature on the wear properties of different PLA-based materials, including the overall influence of moisture on polymers, has been explored, the influence of pressurized hydrous conditions representing more adverse operating conditions, such as deep-water operation or hydraulic systems, on the tribology properties of PLA-based materials during the reciprocating motion has not received due interest. It can be seen that most of the previous explorations entail the evaluation of the influence of individual degradation conditions or ambient conditions for shorter periods of up to 28 days. This research fills an existing knowledge gap by assessing the cumulative influence of hygrothermal aging under pressurized conditions for differing immersion periods and sliding cycles on the tribology properties of 3D-printed PLA and GF-reinforced PLA composites.

The novelty in this study emerges from the integration of a closed-loop, 10 bar high-pressure water-aging process with a comprehensive tribology study performed at various frequencies (0.5 Hz and 1 Hz) and GF weight fractions (0 wt.%, 10 wt.%, and 15 wt.%). In this context the holistic methodology correlates the diffusion kinetics of water, characterized by the use of the Fickian diffusion model parameters, with the trends in the coefficient of friction (COF), wear scars (characterized using 3D laser profilers), and material properties. Following the holistic methodology, the paper explains the highly intricate relationship between fiber loading, the plasticizing effects of aging, interfacial adhesion, and wear mechanisms. The results have large practical applications in various engineering scenarios where the 3D-printed polymer components are simultaneously subjected to mechanical stress and humid, pressurized conditions. These applications include underwater robotics, marine equipment, hydraulic seals, and offshore wind farms.

## 2. Materials and Methods

### 2.1. Filament Fabrication Process

PLA with the trade name “Ingeo™ Biopolymer 4043D” purchased from NatureWorks LLC (Minnetonka, MN, USA) was used as the matrix phase of the polymeric composite filament material used in this study. According to the manufacturer’s datasheet, Ingeo 4043D has a melt flow rate (MFR) of 6 g/10 min (measured at 210 °C/2.16 kg), a relative viscosity of 4.0 (measured in a 1% solution of chloroform, according to ASTM D5225 [37]), and a specific gravity of 1.24 (ASTM D792 [38]). Milled glass fiber was used as the reinforcement phase. Milled glass fiber commercially known as Omfil^®^ was obtained from Omnis Kompozit (Istanbul, Türkiye). According to the supplier’s technical datasheet (FIL 200F), the average fiber diameter is ~13 µm and the characteristic length is ~200 µm. Before composite filament production, both PLA and glass fiber were dried in a vacuum oven at 80 °C for 4 h to remove the moisture in their structures. After drying, mixtures containing glass fiber at 10% and 15% weight ratios were prepared. In the mixture preparation stage, static dry mixing was used to ensure homogeneous dispersion of glass fibers in the PLA matrix during filament extrusion. Through this process, which is carried out by mechanically mixing PLA granules and glass fibers in a closed container, an attempt is made to prevent the glass fibers from agglomerating by ensuring that the glass fibers adhere to the surfaces of the PLA pellets by electrostatic forces. The success of this process was evaluated and verified by optical microscope analysis of PLA pellets randomly selected at the end of the process.

**Figure 1:**
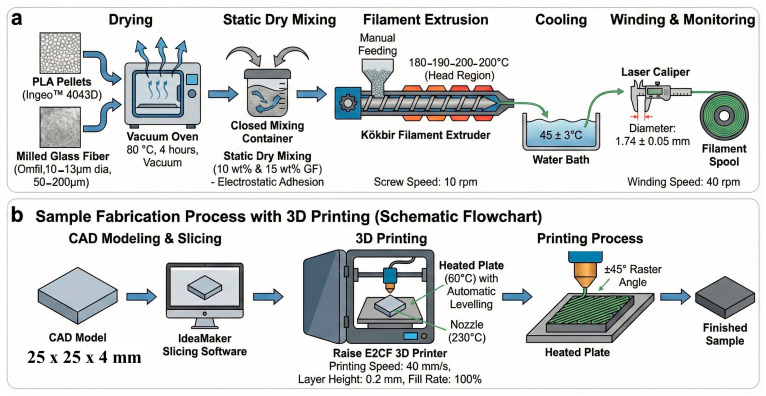
Schematic of the experimental production workflow: (**a**) filament production process including raw material drying, static mixing and controlled extrusion; (**b**) CAD design, slicing and 3D printer sample production stages performed using specified parameters and a ±45° scanning angle.

Using Kökbir brand filament extruder (Kökbir İthalat İhracat Kırklareli/Türkiye), filament fabrication was carried out for three different sample types including neat PLA and glass-fiber-reinforced PLA matrix composite filament compositions with 10 wt.% and 15 wt.% glass fiber reinforcement. The system used for this purpose is a laboratory-type single screw extruder (Kökbir İthalat İhracat Kırklareli/Türkiye), with a screw diameter of approximately 25 mm and an L/D (length/diameter) ratio of approximately 28. A specially modified screw with a high shearing effect was used to ensure more efficient and homogeneous distribution of glass fiber in the PLA matrix. There are four heating zones positioned along the screw and a separately controlled die zone. During the process, critical production parameters such as screw rotation speed, torque, pressure and temperature were monitored in real time via a digital control panel to ensure process stability. The prior preparation of the mixture by electrostatic dry mixing method also supported the glass fiber to adhere to the PLA granules superficially and prevented agglomeration during extrusion and supported the homogeneity of the mixture. The filament fabrication process consists of five series of steps, namely feeding, heating, melting, cooling and winding of the raw material, respectively. The process started with the manual feeding of the prepared mixtures into the feed hopper of the filament extruder, continued with a screw rotation speed of 10 rpm along the temperature profiles of the extruder set as 180–190–200–200 °C (head region), the melted filament was cooled in a water bath of 45 ± 3 °C at the extruder outlet and the winding process was carried out with a pulling speed of 40 rpm. During the extruding/winding process, the filament diameter was continuously monitored with a laser caliper integrated into the extruder to ensure that it was 1.74 ± 0.05 mm (Figure 1a).

### 2.2. Sample Fabrication Process with 3D Printing

3D-printed sample fabrication using filament types in three different compositions was carried out using Raise brand E2CF (Irvine, CA, USA) model 3D printer. CAD models of the specimens (25 × 25 × 4 mm^3^) with geometries according to the characterization tests to be performed were sliced using IdeaMaker software (version 4.3.2). The printing parameters used in sample production were set as 230 °C nozzle temperature, 60 °C plate temperature, 40 mm/s printing speed, 0.2 mm layer height and 100% fill rate. Wear specimens were printed with a raster angle of ±45° to ensure isotropy in their microstructures and to obtain material performance independent of the test direction. All parts were printed directly on the printing table and no support structure was used. In addition, the heated plate used in conjunction with the automatic leveling system ensured that the first layer adhesion was not compromised and the prints were completed with high repeatability (Figure 1b).

### 2.3. Wear Tests

**Figure 2:**
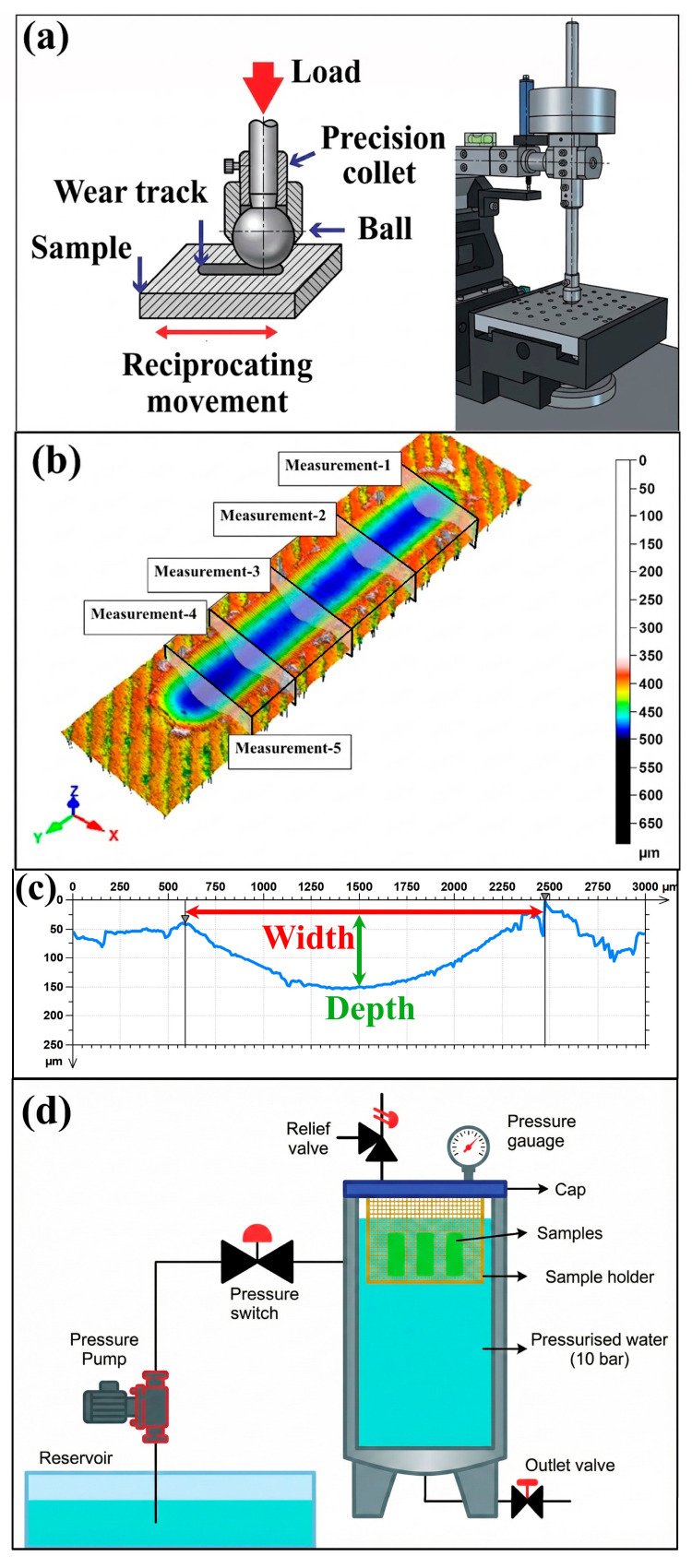
Experimental setup: (**a**) schematics of wear test, (**b**) 3D measurements of wear tracks, (**c**) wear track measurements, (**d**) schematics of pressurized water aging unit.

Wear tests were performed using a UTS Tribolog™ Multi-Function Tribometer (Istanbul, Türkiye), as schematically shown in Figure 2a. During the tests, a stationary 100Cr6 bearing steel ball with a diameter of 6 mm was compressed against the flat surface of the specimen under a 40 N load. A linear reciprocating motion was applied to the specimen, resulting in a wear track on the specimen. The tests were performed at reciprocating frequencies of 0.5 and 1 Hz. The stroke length and number of cycles were adjusted to satisfy the relation 2 × stroke × cycles = 100 m, thereby maintaining a constant total sliding distance of 100 m for all test conditions which are shown in Table 1. Specimens in unaged condition (0 days) and water-aged samples for exposure periods of 7, 14, 21, and 28 days were considered for testing. The tests were performed using a fresh wear track in each test run to avoid any interference due to wear tracks generated in previous tests. The tests were preceded by 24 h exposure of all test samples at 23 ± 2 °C temperature and 50 ± 5% relative humidity conditions. The friction coefficient data was recorded with respect to the distance slide using the data acquisition system available in the tribometer. The wear tracks obtained after testing were examined for a comparative study of weight fraction of glass fiber, frequency of oscillation, and duration of water aging of the specimen on wear response of the composite materials.

### 2.4. Surface Characterization

The post-test surface topography of the reciprocating wear tracks on the 3D-printed PLA composite samples was assessed by a non-contact laser profilometer (Nanovea PS-50, NANOVEA, Irvine, CA, USA). The chosen optical surface topography measurement approach does not involve direct contact between the sensor and the surface of the test sample, ensuring the three-dimensional analysis of the formed wear area and the measurement of the corresponding surface roughness parameters. The obtained topographic images of the samples’ surfaces were analyzed using the Mountains map analysis software (application only version 6.2.7487), and the resulting 3D areal surface topography parameters were analyzed in consideration of the guidelines provided by the international standard ISO 25178 [39]. As schematically illustrated in Figure 2b, the wear scar was scanned and quantified at five distinct locations along each wear track (Measurement 1 to Measurement 5) in order to capture potential spatial variability in the worn topography. For each test condition listed in Table 1, the wear scars were obtained from the corresponding cross-sectional profiles (Figure 2c), where the width was defined by the lateral distance between the two track edges and the depth was taken as the maximum vertical material loss relative to the adjacent unworn surface. For each wear track, five independent measurements were taken in both the transverse (width) and vertical (depth) directions. The final wear scar width and depth values reported in this study correspond to the arithmetic average of these five individual measurements.

### 2.5. Water Immersion Procedure

The hydrothermal aging was conducted at a constant pressure of 10 bar to facilitate an accelerated testing environment. This pressure level enhances the chemical potential of water, thereby increasing the diffusion rate into the PLA matrix and along the glass fiber interfaces. According to the literature, such pressurized conditions are effective in simulating long-term environmental exposure in a condensed timeframe by accelerating moisture-induced degradation mechanisms like plasticization and interfacial debonding, without altering the fundamental failure modes of the composite.

The high-pressurized water aging rig, shown schematically in Figure 2d, consists of a closed hydrostatic loop using a high-pressurized pump that sucks water from an outside tank, as well as a pressure switch that allows variation in the flow rate in the line. Aging trials were performed in a closed, leak-tight steel tank, where the internal pressure was permanently recorded using a manometric gauge embedded in the tank cap, maintained at a constant level of 10 bar over the entire duration of the aging tests. To allow for simultaneous exposure of the specimens from all sides, as well as to avoid contact between specimens, the specimens were placed in a porous sample holder (or cage) that ensures complete immersion of the specimens. Safety measures in the experimental setup include a safety valve on the tank, in addition to an outlet valve located at the bottom of the tank, enabling control of the drainage of the water after the completion of each experimental session.

In the current investigation, the water uptake of pure PLA and glass-fiber-reinforced composites with dimensions 25 × 25 × 4 mm were studied with fiber fractions of 10% and 15%, respectively, at a hydrostatic pressure of 10 bar. To ensure the accuracy and repeatability of the results, the water uptake tests were performed in triplicate (*n* = 3) for each category. The average values were recorded, and the standard deviations were calculated for statistical analysis.

### 2.6. Differential Scanning Calorimetry (DSC) Analysis

Thermal characteristics of 3D-printed neat PLA and glass-fiber-reinforced PLA composite specimens, both in unaged condition and after hydrolytic aging, glass transition temperature (Tg), cold crystallization temperature (Tcc), and melting temperature (Tm), as well as the corresponding cold crystallization enthalpy (ΔHcc) and melting enthalpy (ΔHm), were determined using a TA Instruments Q200 DSC system manufactured by TA Instruments Inc. (New Castle, DE, USA). All DSC measurements were carried out in the temperature range of 30–200 °C at a constant heating rate of 5 °C/min.

### 2.7. Scanning Electron Microscopy (SEM) Analyses

The wear surface morphologies of 3D-printed pure PLA and GF-reinforced PLA matrix composite samples with and without hydrolytic aging were analyzed by scanning electron microscopy (SEM). For this purpose, JEOL brand JCM-6000 model SEM device (JEOL Ltd. (Tokyo, Japan)) was used. Before the analysis, the surfaces of the polymeric samples were gold coated to provide electrical conductivity. The coating process was carried out in a vacuum environment of 10^−2^ bar, with a discharge current of 30 mA and a coating time of 120 s.

## 3. Results

### 3.1. Water Uptake Behavior

The kinetics of water absorption were evaluated based on the one-dimensional Fickian diffusion equation. The diffusion coefficients D and the corresponding saturated mass M∞ were calculated from the initial slope of the Mt vs. √t graph and are succinctly discussed in Table 2 below.

Analyzing the diffusion parameters given in Table 2, the effect of reinforcement from the glass fiber (GF) on the water absorption behavior of the composite material can be identified. From the results, the reinforcement from the GF causes a substantial increase in the saturated water mass. While the equilibrium water mass for the pure PLA was 0.0845 g, it increased to 0.1625 g for 10% GF-reinforced materials and to 0.2950 g for 15% GF-reinforced materials, an increase of about two times and three and a half times, respectively. The above observations confirm that the hydrophilic properties as well as higher volume of glass fibers will result in an increase in the water storage behavior.

Comparisons of the calculated diffusion coefficients (D) show that the values are very closely spaced, with a range of 4.31 × 10^−12^ to 4.52 × 10^−12^ m^2^/s. However, a slight increasing trend of the value of D with the increase in fiber concentration can be noted. This indicates that the rate of penetration of water (kinetics) into the material is mainly affected by the 10 bar hydrostatic pressure and PLA matrix. However, the increase in the value of D indicates that the diffusion paths along the fiber/matrix interfaces assist the transportation of the water molecules.

**Figure 3:**
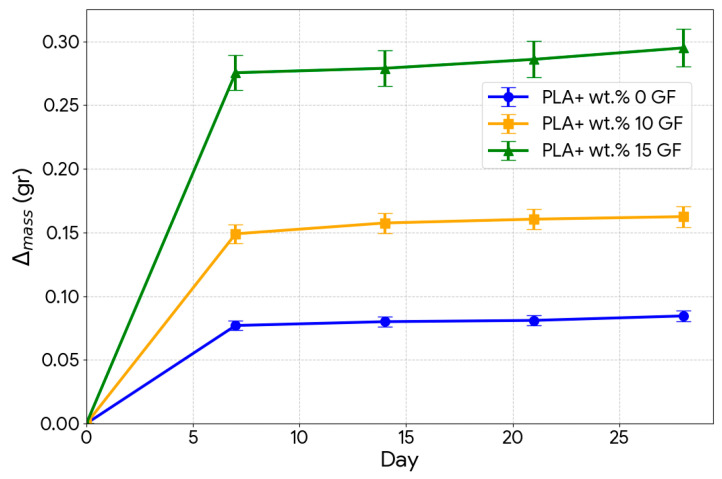
Mass change (Δmass) of neat PLA and GF-reinforced PLA composites (10 wt.% and 15 wt.%) versus immersion time under 10 bar hydrostatic pressure (Error bars represent a standard deviation within a 3–5% range based on triplicate measurements).

Figure 3 and Table 2 demonstrate an increase in glass fiber reinforcement ratio (from 0 wt.% to 15 wt.%) which improves the water uptake. This is due to the inherent properties of materials used in the formulation of this biodegradable composite. The PLA matrix used in this study is less absorbent in nature due to its naturally hydrophobic properties (semi-crystalline in structure as well as an ester-based polymer), while glass fibers in this study are naturally more hydrophilic due to their inherent properties of surfaces rich in silanol group (Si-OH) units as well as polar oxide materials. The augmented number of polar sites in fibers from 10 wt.% to 15 wt.% enhances the overall ability to retain water in this biodegradable polymer. The increased concentration of fibers also aids in creating an inorganic framework in this biodegradable polymer matrix. Under high hydrostatic pressure, such as 10 bar, water molecules tend to move not only between the polymer chains but also along the fiber surfaces (wicking effect). Increasing the fiber volume ratio expanded the surface area through which water could move within the material, thus supporting the diffusion process. Although the diffusion coefficients (D) are similar, the saturation mass of the 15% GF-added sample is approximately 3.5 times that of pure PLA. This indicates that the rate at which water penetrates the material (kinetic) is dominated by pressure; however, the amount of water retained (capacity) is directly controlled by the proportion of hydrophilic components in the material (fiber ratio). Hence, the increase in glass fiber content contributed to the increment in the chemical affinity of the material to water, leading to the absorption of water under pressure. Notably, the markedly higher moisture capacity of the 15 wt.% GF composite provides a mechanistic basis for the non-monotonic friction response discussed in Section 3.2, particularly the reduced COF observed at 21-day aging, which is interpreted as an intermediate state between matrix plasticization and extensive interface-controlled damage.

### 3.2. COF Examinations

**Figure 4:**
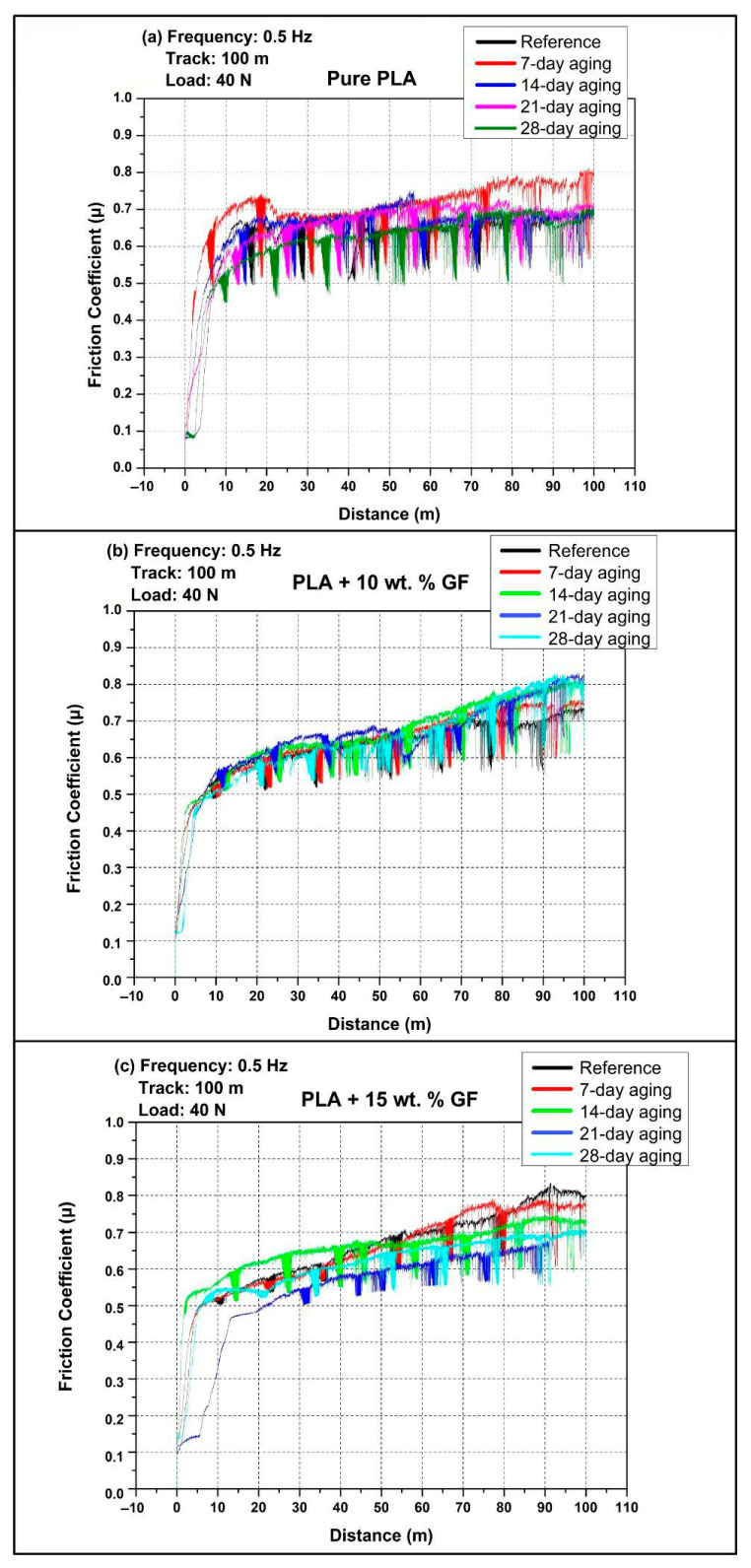
Effect of water aging time on COF values at 40 N load, 100 m track at 0.5 Hz: (**a**) pure PLA; (**b**) PLA + 10 wt.% GF; (**c**) PLA + 15 wt.% GF.

Figure 4 shows the friction coefficient (COF) stages during reciprocating wear at 40 N, 0.5 Hz, and a total track length of 100 m for three 3D-printed PLA-based systems. In line with the experimental plan shown in Table 2, the tribological response of the PLA-based systems was evaluated in both the as-processed (non-aged) state and following water immersion periods of 7, 14, 21, and 28 days. For all material configurations, the friction curves display an initial brief running-in phase, which is subsequently followed by a more extended steady-state regime where COF fluctuates around a characteristic level, with intermittent sharp drops that indicate transient instabilities of the contact, such as momentary loss and reformation of the transfer layer or stick slip events.

For Figure 4a (pure PLA), the reference specimen rapidly increases from a low initial COF to a quasi-steady level around 0.65 to 0.70 within the first roughly 10 to 20 m, then remains broadly stable with moderate fluctuations up to 100 m. The 7-day-aged PLA shows the highest friction response among the aging conditions, with an early overshoot to roughly 0.70 to 0.73 and a progressive increase after about 60 m, reaching about 0.75 to 0.80 toward the end of the track. The 14-day- and 21-day-aged curves largely overlap the reference in the mid distance range, typically stabilizing around 0.66 to 0.70, with the 14-day condition showing a local rise near the mid-track where COF momentarily approaches the low 0.70 s. The value of the 28-day-aged PLA is seen to be marginally lower than that of other ages for most of the period, ranging roughly between 0.63 and 0.68, which reflects a relatively lower level of friction. This is in spite of the fact that their initial variations are similar.

For Figure 4b (PLA + 10 wt.% GF), the initial running is again rapid, but the subsequent behavior is characterized by a more gradual, distance-dependent increase in COF. In the first 10 to 20 m, COF typically rises to about 0.50 to 0.60, then continues increasing slowly through the mid-track. Beyond about 60 m, the curves diverge more clearly with aging time. The reference condition remains the lowest overall, ending at about 0.70 to 0.73 at 100 m. Water-aged specimens generally show elevated late-stage COF, with the 7-day condition ending around the mid-0.70 s, while the 14-day and 28-day conditions reach the highest terminal values, approaching about 0.80 to 0.82. The 21-day curve is typically intermediate, rising above the reference after mid-track and finishing around the high 0.70 s. Across all aging states, frequent short downward excursions are visible, but the dominant trend for this composite is that longer immersion tends to amplify the late track friction increase.

Considering the composite type effect across Figure 4, the friction response transitions from a comparatively flat, plateau-like behavior in pure PLA to a more distance-dependent increase when glass fiber is introduced. Pure PLA mostly clusters around 0.65 to 0.70 after running in, except for the 7-day-aged condition that rises to about 0.75 to 0.80 late in the test. The 10 wt.% GF composite shows the most consistent late-stage COF escalation with aging, reaching the highest end of track values overall, up to about 0.80 to 0.82 for longer immersion. The 15 wt.% GF composites can either match the high friction levels of the 10 wt.% GF system in the reference and 7-day states or deliver a lower sustained COF under specific aging conditions, most clearly at 21-day where it becomes the lowest friction case among the reinforced materials. Overall, adding GF increases the sensitivity of COF to water aging and sliding distance, while increasing GF content from 10 wt.% to 15 wt.% does not uniformly increase COF, instead yielding aging-dependent shifts in both the running in behavior and the steady friction level.

**Figure 5:**
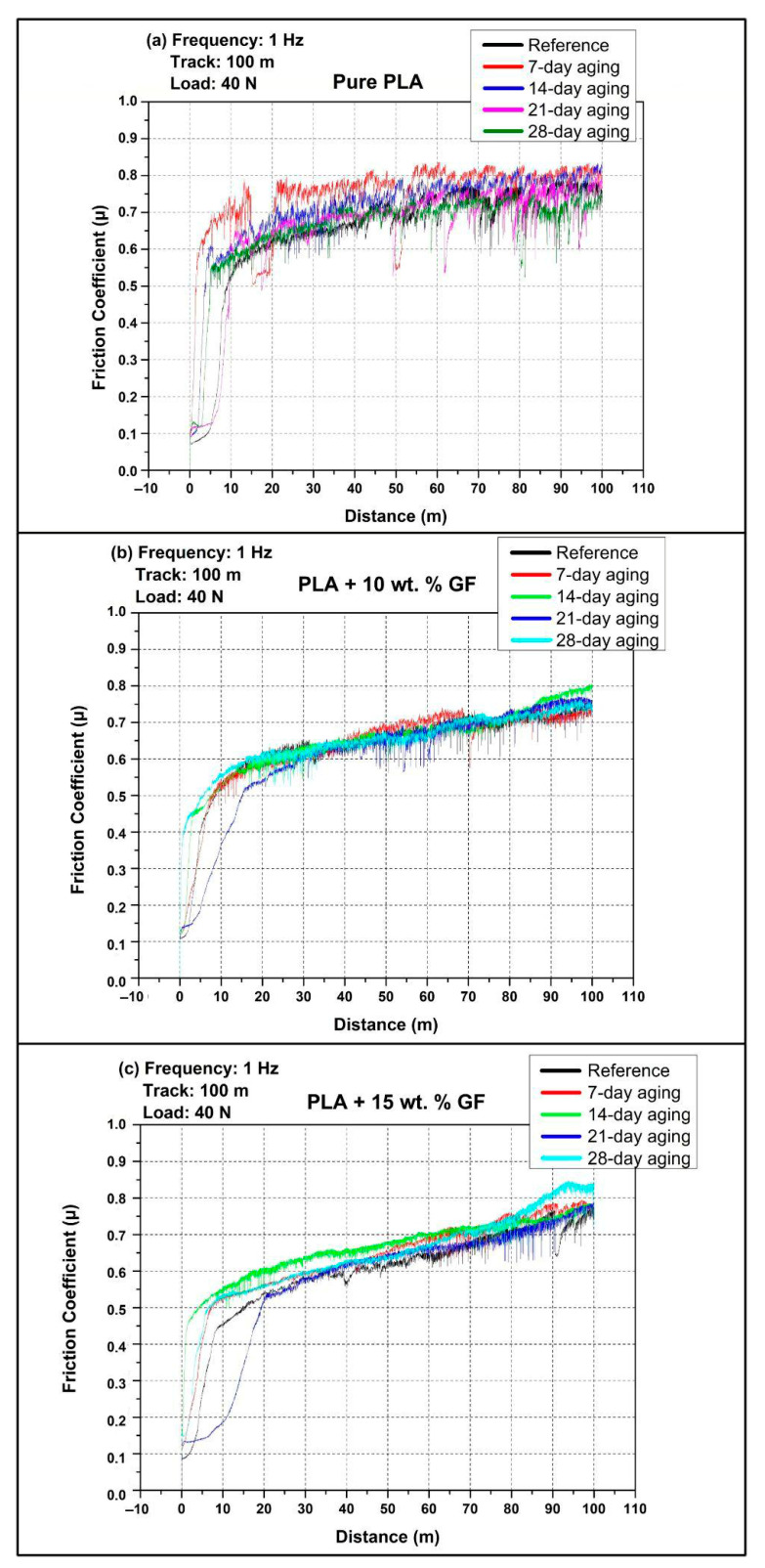
Effect of water aging time on COF values at 40 N load, 100 m track at 1 Hz: (**a**) pure PLA; (**b**) PLA + 10 wt.% GF; (**c**) PLA + 15 wt.% GF.

Figure 5 shows the friction coefficient (COF) stages with sliding distance during 1 Hz reciprocating wear under 40 N over a 100 m track, comparing the reference condition (no aging) with 7-day, 14-day, 21-day, and 28-day water immersion, in accordance with the aging matrix in Table 2. Across all three panels, the COF curves exhibit a short running in period in the first few meters, followed by a longer regime where friction gradually increases with distance and is interrupted by occasional sharp drops, which are indicative of transient contact instabilities such as local disruption and re-establishment of the polymer transfer layer, intermittent debris entrainment, or short stick slip events.

In Figure 5a (pure PLA), all conditions rapidly transition from an initially low COF to a higher steady sliding regime within the first roughly 5 to 15 m. After running in, the reference specimen typically stabilizes around approximately 0.62 to 0.70 in the early to mid-track and then shows a gradual increase, reaching about 0.78 to 0.82 toward 100 m. Water aging generally shifts the pure PLA curves upward and amplifies fluctuations. The 7-day-aged specimen shows the most pronounced early COF rise, rapidly reaching roughly 0.70 to 0.75 and maintaining the highest friction band over much of the test, often around approximately 0.75 to 0.82. The 14-day and 28-day curves largely overlap the reference during the mid-track but remain slightly higher at long distances, typically ending close to about 0.80. The 21-day-aged curve tends to remain comparatively lower than the other aged conditions for most of the track, usually clustering around approximately 0.65 to 0.75 and ending below the highest curve group, although still showing an overall distance driven increase.

In Figure 5b (PLA + 10 wt.% GF), the COF responses are more tightly clustered than pure PLA, indicating that the addition of glass fiber reduces the sensitivity of friction to aging time under 1 Hz, at least within the scatter of the signals. After the initial running in, all conditions converge to a similar friction level in the range of about 0.55 to 0.62 by roughly 10 to 20 m, followed by a nearly monotonic, slow increase with distance. The reference condition generally occupies the lower envelope through the later stages, ending around approximately 0.72 to 0.75 at 100 m. The 7-day- and 14-day-aged conditions are typically slightly higher than the reference across the mid-to-late track, often ending around 0.74 to 0.77. The 21-day- and 28-day-aged specimens show the highest terminal COF values within this composite family, approaching approximately 0.77 to 0.79 near the end of the track. Overall, the dominant feature is the gradual distance-dependent increase rather than strong separation by immersion duration.

In Figure 5c (PLA + 15 wt.% GF), the curves show clearer differentiation with aging time, particularly in the late track. Following running in, friction values generally lie around approximately 0.50 to 0.60 in the early to mid-distances and then increase progressively. The reference condition remains comparatively lower over much of the track and typically finishes around approximately 0.75 to 0.78, with intermittent sharp drops near the end suggesting occasional loss of stable sliding. The 7-day-aged condition trends higher than the reference during the second half of the test and reaches about 0.78 to 0.80 at 100 m. The 14-day- and 21-day-aged curves are usually intermediate, tracking close to each other for most of the distance and ending around approximately 0.76 to 0.79. The 28-day-aged specimen exhibits the most prominent late-stage change, with an evident turnaround after a distance of 80–90 m, and a maximum terminal value of the COF of around 0.82–0.85. This implies that the immersion for a longer period of time has the most significant friction-escalating effect in a scenario of increased GF concentration, namely 15 wt.%, and at a high frequency of sliding.

Comparing composite types at 1 Hz, pure PLA generally exhibits the highest and most fluctuating friction response, with steady sliding typically evolving into a high COF band near approximately 0.78 to 0.82 by the end of the track, and with water aging, especially 7-day, further promoting higher friction throughout much of the test. Introducing GF produces more clustered and smoother COF trajectories and shifts the behavior toward a gradual, distance-dependent increase. The PLA + 10 wt.% GF system shows the smallest separation among aging durations and maintains terminal COF values mostly below pure PLA, typically around approximately 0.72 to 0.79. Increasing reinforcement to 15 wt.% GF strengthens the effect of long immersion time, with 28-day aging producing the highest end of track COF (about 0.82 to 0.85) among the reinforced composites, while shorter aging durations remain closer to the reference. Overall, at 1 Hz the influence of water aging on friction becomes more pronounced at higher GF content, primarily through elevated late-stage COF rather than major changes in the initial running in level.

As illustrated in Figure 3 and Figure 4, the observed coefficient of friction (COF) is consistent with the characteristic reciprocating wear behavior reported for polylactic acid (PLA) and PLA-based composite systems. The findings presented in this study are consistent with previously published research on the tribological behavior of PLA-based materials produced using FDM. Earlier studies have reported that modifications such as additive incorporation, filler reinforcement, or structural tailoring affect the friction coefficient and wear mechanisms of PLA composites under sliding conditions. The general trends observed in the present work—particularly the influence of reinforcement content on frictional response, wear development, and material–counterface interaction—are in agreement with these studies, confirming that the tribological behavior identified here aligns with the established literature [40,41,42,43]. This phenomenon explains the COF behavior in reinforced PLA, in which high levels of COF result because of fluctuations in COF as influenced by third-body wear, transfer film degradation, and surface damage.

### 3.3. Wear Damage Evaluation

**Figure 6:**
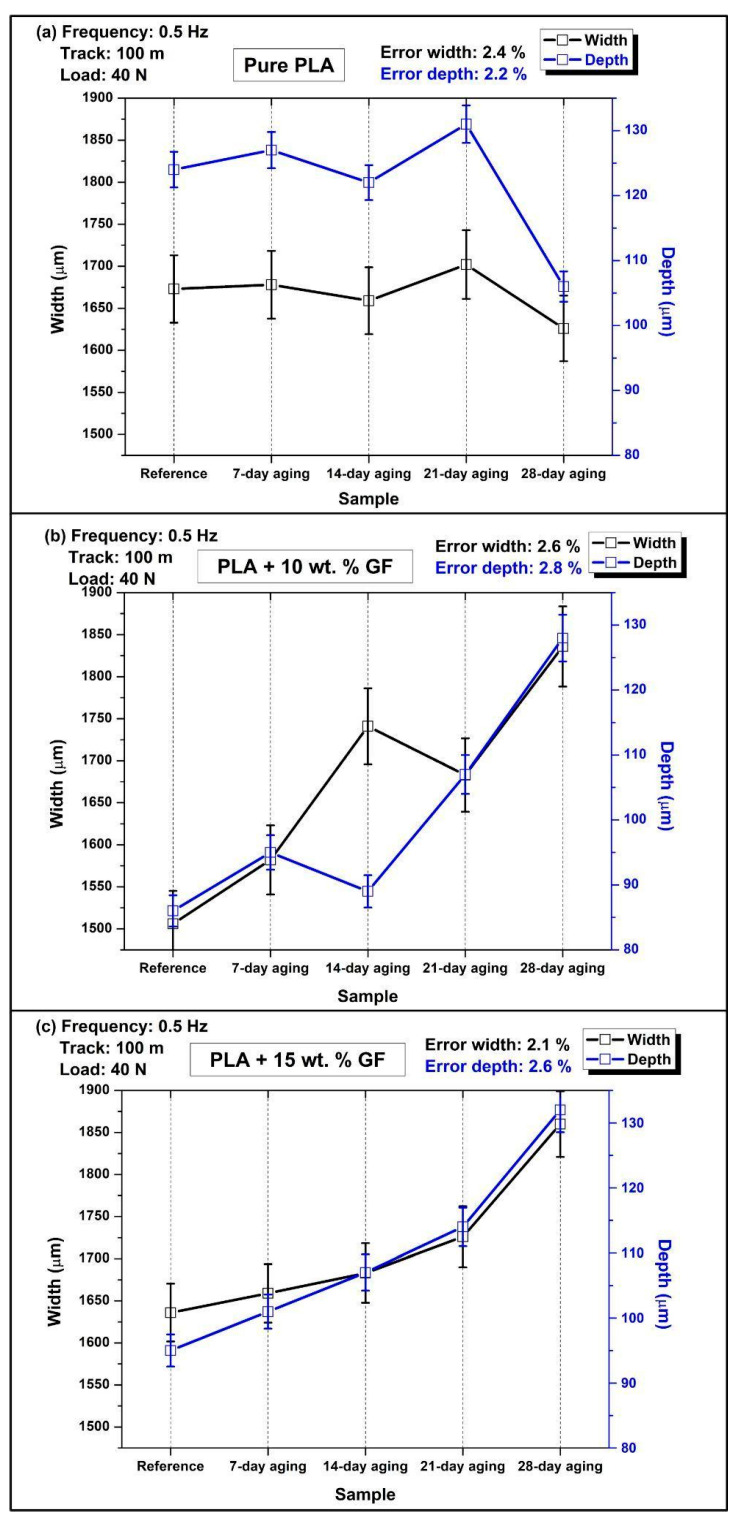
Wear scar widths and depths under a normal load at 40 N load, 100 m track at 0.5 Hz: (**a**) pure PLA; (**b**) PLA + 10 wt.% GF; (**c**) PLA + 15 wt.% GF.

Figure 6 compares the wear scar width (left axis, black markers) and the maximum wear scar depth (right axis, blue markers) measured after reciprocating sliding at 40 N, 0.5 Hz, and of 100 m distance for pure PLA and glass-fiber-reinforced PLA (10 wt.% and 15 wt.% GF). As shown in the water immersion matrix in Table 2, the baseline (no aging) is contrasted with samples aged in water for 7-day, 14-day, 21-day, and 28-day. These points show the mean level for each condition with error bars showing the repeatability of the measurement. The reported measurement uncertainty is low for all cases, with error widths of 2.4% to 2.6% and error depths of 2.2% to 2.8%, indicating that the observed differences among aging durations are systematic rather than within experimental scatter.

In the case of Figure 6a, which corresponds to pure PLA, the width of the wear scar remains within a short range for different aging periods ranging from around 1625 µm to 1700 µm. The width of about 1670 µm and the corresponding depth of about 125 µm are seen in the reference sample. After 7 days of water aging, the dimensions remain almost the same (width of about 1675 µm) with a slight increase in the depth (around 127 µm), signifying slightly increased wear. The dimensions are slightly smaller in the 14-day-aged sample (width of about 1660 µm and depth of about 123 µm), possibly signifying the stabilization process of the contact area with a short-term changeover in the transfer film or maybe also associated with surface restructuring. The maximum dimensions (both width and depth) are measured in the 21-day water-aged sample with corresponding dimensions of about 1700 µm and 131 µm. These show increased wear. It can be noted that the dimensions for the 28-day water-aged pure PLA are smaller with a width of about 1625 µm and a significantly smaller depth of roughly 107 µm.

In Figure 6b, for PLA with 10 wt.% glass fibers (PLA + 10 wt.% GF), the effect of water aging shows an ostensibly higher degree of dispersion in wear parameters than in pure PLA, especially at longer immersion periods. In the unaged configuration, the combined material shows the lowest dimensions in the wear tracks, with a width of about 1510 µm and a depth of approximately 85 µm, which reflects the efficacy of reinforcement in hindering penetration and minimizing material loss at 0.5 Hz. At 7-day, the width and depth increase to about 1585 µm and 95 µm, respectively, as expected for the initial stages of moisture-driven degradation of the polymer properties and fiber/matrix interfaces. At 14-day, the width of the wear tracks markedly increases to about 1740 µm, with a corresponding decrease in the depth to approximately 88 µm, which indicates relative material loss that is largely lateral and confined to widening the track rather than substantial gouging, possibly related to microcracking, fiber exposure, and gentle plowing actions in this stage. At 21-day, the depth shows a substantial increase to about 108 µm, with a corresponding slight reduction in width to about 1685 µm, which indicates a transition to a more penetrative form of material loss. The most degraded stages are at 28-day, with both width and depth reaching their highest levels at approximately 1830 µm and 129 µm, respectively, which recalls the cumulative effects of moisture-driven interfacial unbonding, possibly accelerating fiber pull-out or debonding with enhanced third-body abrasion action to widen the tracks and increase their depth.

For Figure 6c (PLA + 15 wt.% GF), both wear scar width and depth increase almost monotonically with water aging time, showing the strongest time dependence among the three material systems. The reference specimen starts with a width of approximately 1635 µm and a depth near 96 µm, already deeper than the 10 wt.% GF references but still lower than the pure PLA reference depth. After 7-day, width and depth rise slightly to about 1660 µm and 101 µm, then continue increasing at 14-day (about 1685 µm width, 108 µm depth) and 21-day (about 1725 µm width, 115 µm depth). The condition of the 28-day-aged sample shows a pronounced increase, reaching around 1860 µm in width and 132 µm in depth. The monotonically increasing curve suggests that higher amounts of fibers could increase the susceptibility to the effects of longer immersion times, primarily due to the additional interfaces and the possibility of capillary paths which could facilitate faster invasion of water, thus leading to more pronounced removal of materials.

Across composite types, the reference condition indicates that GF reinforcement can reduce wear depth substantially relative to pure PLA, decreasing from approximately 125 µm in pure PLA to about 85 µm for 10 wt.% GF and about 96 µm for 15 wt.% GF, while also reducing or maintaining comparable track widths depending on GF content. However, under prolonged water aging the ranking reverses. At 28-day, pure PLA shows the smallest depth, approximately 107 µm, and the smallest width, about 1625 µm, whereas both GF-reinforced systems exhibit considerably larger scars. The 10 wt.% GF and 15 wt.% GF specimens reach widths of approximately 1830 µm and 1860 µm, with depths of about 129 µm and 132 µm, respectively. Therefore, at 0.5 Hz and 40 N, GF additions are beneficial in the unaged state but lead to greater wear sensitivity after extended immersion, with the 15 wt.% GF composites showing the most pronounced time-driven escalation in both widening and penetration.

**Figure 7:**
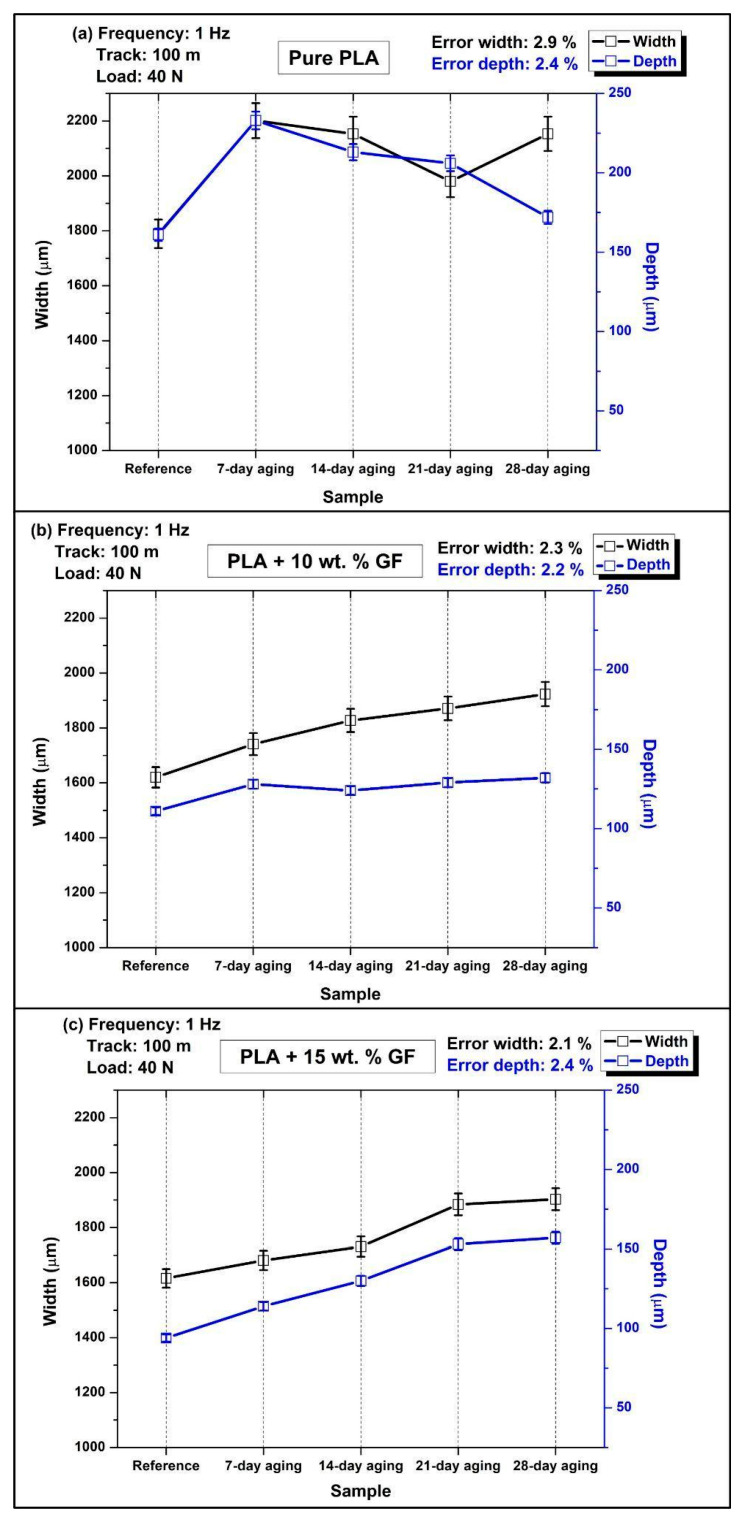
Wear scar characteristics as a function of water-aging duration for neat PLA and glass-fiber-reinforced PLA composites, at 1 Hz under a normal load of 40 N with a total sliding distance of 100 m: (**a**) neat PLA; (**b**) PLA + 10 wt.% GF; (**c**) PLA + 15 wt.% GF.

Figure 7 shows the variation in wear scar width (left *y*-axis) and maximum wear scar depth (right *y*-axis) for neat PLA and glass-fiber-reinforced PLA composites containing 10 wt.% and 15 wt.% GF, determined from reciprocating wear tests performed under a normal load of 40 N at 1 Hz with a total sliding distance of 100 m. The results are presented as a function of water immersion duration (unaged, 7, 14, 21, and 28 days), in accordance with the aging scheme outlined in Table 2. The comparatively low measurement uncertainties reported on each panel, error width 2.1% to 2.9% and error depth 2.2% to 2.4%, indicate that the observed aging-driven shifts in wear metrics are meaningful and not dominated by experimental scatter.

In Figure 7a (pure PLA), water aging strongly increases the penetration component of wear, particularly at short to intermediate immersion times. The reference depth is approximately 165 µm, whereas the 7-day-aged condition reaches the maximum depth, approximately 235 µm to 240 µm, indicating a pronounced loss of near-surface load bearing capacity after early water exposure. On further immersion, the depth keeps reducing, and at 14-day, the depth is about 210–215 µm, at 21 days about 205–210 µm, and at 28-day the depth is approximately 175 µm, which shows the partial recovery of the resistance to deep grooving. The width values among the aged pure PLA conditions remain high overall, peaking at about 2200 µm at 7-day, decreasing to about 2140 µm at 14-day, reaching the minimum around 1980 µm at 21-day, and increasing again to about 2140 µm at 28-day. This combination, very high depth at 7-day with simultaneously large width, is consistent with severe adhesive plus abrasive damage when the matrix is most susceptible to moisture-induced softening or hydrolytic weakening, while the reduced depth at 28-day together with re expanded width implies a shift toward more laterally distributed material removal and/or a more stable but broader tribo-layer response rather than purely penetrative plowing.

As shown in Figure 7b for the PLA composite containing 10 wt.% glass fibers, a progressive increase in wear scar width is observed with increasing water-aging duration. The average width rises from approximately 1620 µm in the unaged condition to nearly 1740 µm after 7 days of immersion, followed by further increases to about 1825 µm at 14 days, 1870 µm at 21 days, and reaching roughly 1920 µm after 28 days. The depth changes are comparatively modest relative to pure PLA, rising from about 115 µm (reference) to about 130 µm (7-day), dipping slightly to about 125 µm to 126 µm (14-day), then increasing again to about 130 µm (21-day) and about 133 µm (28-day). The data indicate that water aging in the 10 wt.% GF composite primarily promotes track broadening, while the depth remains constrained within a narrow band, which is consistent with reinforcement limiting penetration even as matrix softening and progressive interface deterioration facilitate wider damage and debris-assisted lateral abrasion.

For the PLA composite reinforced with 15 wt.% glass fiber (Figure 7c), both the wear scar width and maximum depth increase systematically with prolonged water immersion, with the depth evolution being more pronounced than that observed for the 10 wt.% GF formulation. The wear scar width expands from roughly 1610 µm in the unaged condition to around 1690 µm after 7 days, followed by values of approximately 1730–1740 µm at 14 days, and then shows a marked rise to nearly 1890 µm and 1910 µm after 21 and 28 days of aging, respectively. In parallel, the maximum wear depth increases from about 95 µm in the reference state to approximately 115 µm (7 days) and 130 µm (14 days), before rising more rapidly to around 155 µm at 21 days and reaching nearly 160 µm after 28 days. The step-like increase after 14 days suggests that prolonged immersion increasingly compromises the integrity of the fiber–matrix interphase and promotes more penetrative damage modes, such as fiber debonding, pull-out, and third-body abrasion, which can raise both penetration depth and track widening during reciprocating sliding.

Across composite types, the reference condition confirms the beneficial role of GF addition in reducing penetration, pure PLA shows the highest depth at about 165 µm, PLA + 10 wt.% GF decreases to about 115 µm, and PLA + 15 wt.% GF decreases further to about 95 µm, while the reference widths for both reinforced systems remain around 1610 µm to 1620 µm. Under water aging, pure PLA exhibits the most severe depth excursion at short aging, reaching about 235 µm to 240 µm at 7-day, whereas the reinforced systems remain far lower at the same immersion time, about 130 µm for 10 wt.% GF and about 115 µm for 15 wt.% GF. At prolonged aging, the reinforced systems show continued widening, and the 15 wt.% GF composite in particular shows a strong depth increase up to about 160 µm at 28-day, narrowing the performance gap relative to pure PLA at long immersion. Overall, GF reinforcement improves wear resistance in the unaged state and limits early depth escalation, but extended water exposure progressively increases wear scar width in both composites and promotes deeper penetration at higher fiber content, indicating that moisture sensitivity of the interfacial network becomes a dominant factor at longer aging durations under 1 Hz loading conditions.

The increases in wear scar width and depth observed in Figure 6 and Figure 7 are consistent with previously reported findings on aged fiber-reinforced composite systems. Earlier studies have shown that moisture exposure and hydrothermal aging generally promote matrix softening, interface degradation, and fiber–matrix debonding, all of which contribute to widened and deepened wear tracks under sliding conditions [24,44,45]. The trends identified in the present work—particularly the progressive deterioration of interfacial integrity and the associated changes in wear morphology—are therefore in agreement with the established literature on the tribological behavior of water-aged, fiber-reinforced composites.

### 3.4. Profilometric Analysis of Wear Track Damage

**Figure 8:**
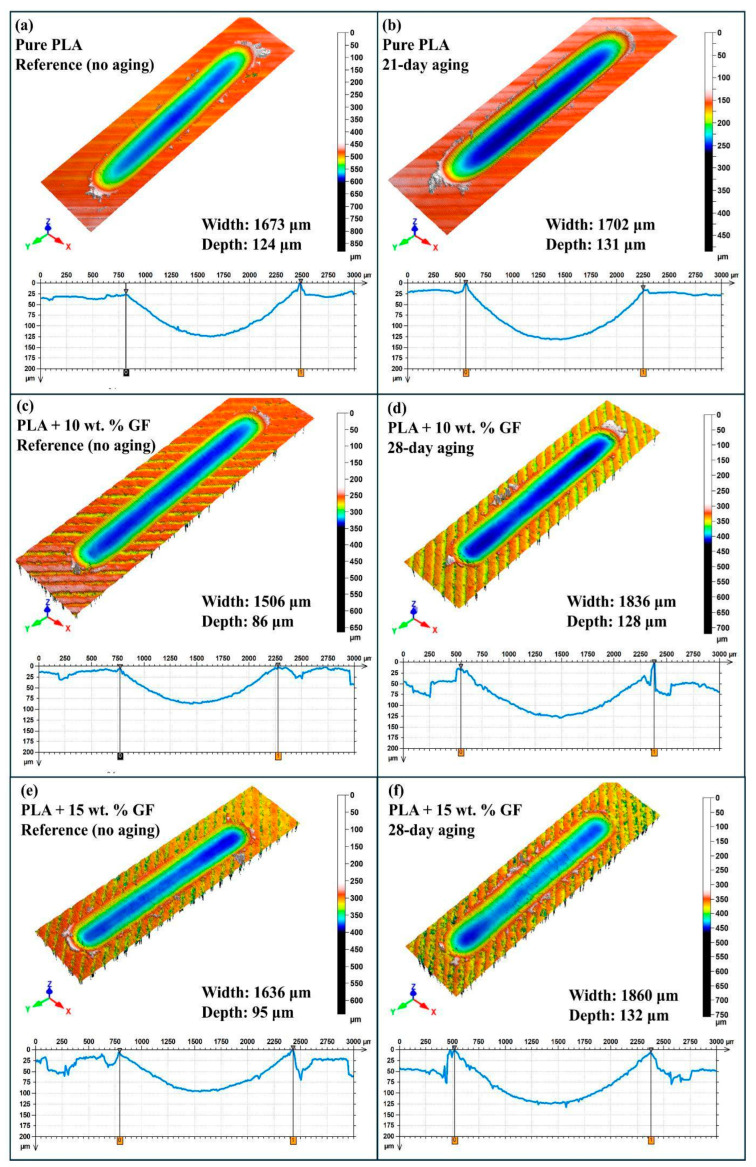
Wear scar width and depth as a function of aging duration at 0.5 Hz under a 40 N load (100 m sliding distance) (**a**) pure PLA, reference; (**b**) pure PLA 21-day aging; (**c**) PLA + 10 wt.% GF, reference; (**d**) PLA + 10 wt.% GF, 28-day aging; (**e**) PLA + 15 wt.% GF, reference; (**f**) PLA + 15 wt.% GF, 28-day aging.

Figure 8 is intended to provide a within-group comparison between the reference condition and the specimen that exhibited the maximum wear scar depth among the tested aging durations for that same material system. Accordingly, the figure does not represent a full chronological sequence for every aging interval. Instead, it highlights the worst-case depth response relative to the corresponding unaged baseline, enabling a direct assessment of the most detrimental aging condition for each formulation under 40 N, 0.5 Hz and 100 m reciprocating sliding.

For pure PLA, the reference specimen in Figure 8a is compared with the aged condition in Figure 8b, which shows the maximum depth within the pure PLA set. The unaged PLA exhibits a wear scar width of 1673 µm and a minimum depth of 124 µm, with a smooth valley profile and moderate pile up at the track edges. Under the aging condition selected as the maximum depth case, the wear scar depth increases to 131 µm and the width to 1702 µm, corresponding to an increase of approximately 5.6 percent in depth and approximately 1.7 percent in width relative to the reference. The deeper penetration and slightly enlarged track indicate that the most severe aging state for neat PLA within the tested window promotes higher subsurface compliance and facilitates greater counterface indentation during reciprocating motion.

For PLA + 10 wt.% GF, Figure 8c presents the reference surface, while Figure 8d corresponds to the aging duration at which the maximum depth was recorded within this composite group. In the unaged condition, the composite shows a reduced wear scar depth of 86 µm and a width of 1506 µm, confirming the beneficial role of glass fiber reinforcement on load support and penetration resistance at the initial state. In the maximum depth aging condition, the depth rises to 128 µm and the width increases to 1836 µm, which corresponds to approximately 49 percent higher depth and approximately 22 percent higher width compared with the reference. The transition from a shallow groove to a markedly deeper valley in the worst-case aged specimen is consistent with aging-induced deterioration of the fiber matrix interface, which weakens the reinforcement efficiency and allows the contact to penetrate more aggressively.

For PLA + 15 wt.% GF, Figure 8e shows the reference condition and Figure 8f shows the aging duration that produced the maximum depth within this group. The unaged 15 wt.% GF composite exhibits a wear scar depth of 95 µm and a width of 1636 µm, which is deeper and wider than the 10 wt.% GF reference, indicating that the higher fiber loading does not further decrease wear penetration in the baseline state, likely due to increased heterogeneity and local stress concentrations. Under the maximum depth aging condition, the depth increases to 132 µm and the width to 1860 µm, corresponding to approximately 38.9 percent higher depth and approximately 13.7 percent higher width relative to the unaged reference. The deeper and broader groove morphology in the worst-case aged specimen supports the interpretation that, within the tested aging window, the most critical degradation state in the 15 wt.% GF composite substantially accelerates penetration and track expansion.

Overall, the within-group comparisons in Figure 8 demonstrate that the aging duration associated with the maximum wear scar depth differs by material system, and the magnitude of the degradation is strongly formulation dependent. Neat PLA shows a relatively modest depth increase at its maximum depth aging condition, whereas both GF-reinforced composites exhibit pronounced increases in depth at their respective maximum depth aging durations, indicating that aging-driven interfacial weakening dominates the wear penetration response once fibers are introduced.

**Figure 9:**
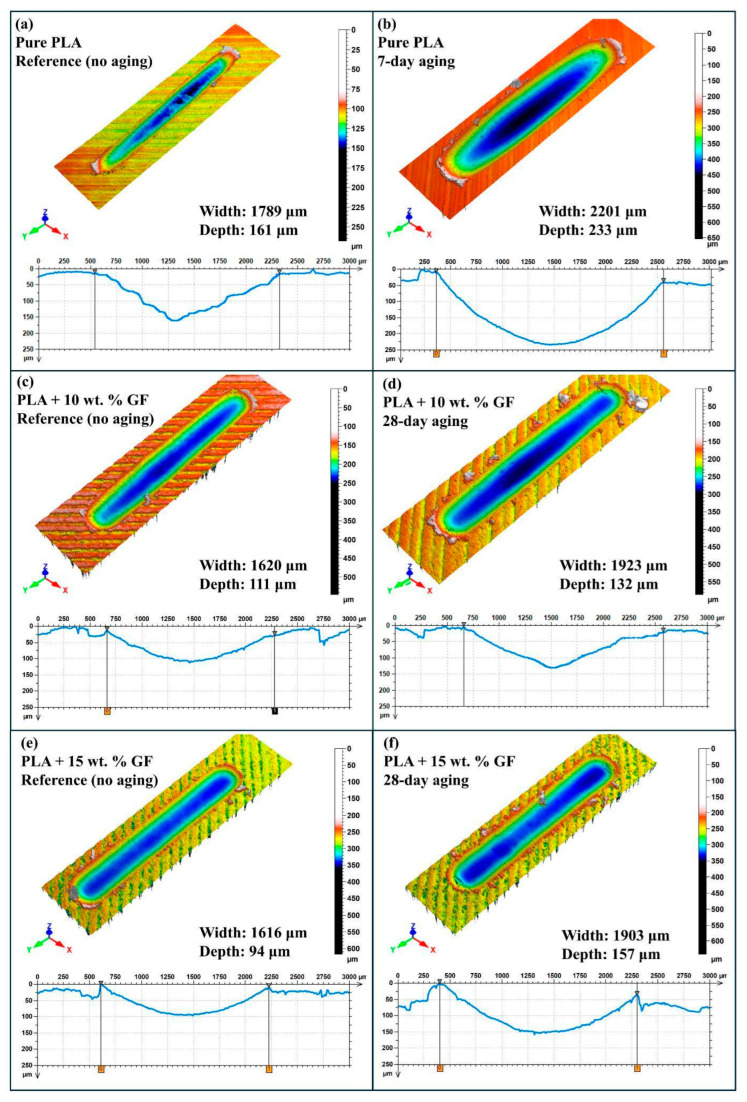
Wear scar width and depth as a function of aging duration at 1 Hz under a 40 N load (100 m sliding distance).

Figure 9 compares the post-test three-dimensional wear scar morphologies and the corresponding transverse profiles for pure PLA and PLA reinforced with 10 wt.% and 15 wt.% glass fibers under reciprocating sliding at 40 N, 1 Hz and a 100 m track length. For each material system, the unaged reference specimen is paired with the aged condition that produced the maximum wear scar depth within that same group. This selection strategy highlights the worst-case penetration behavior driven by water aging, while keeping the baseline unchanged, so that the relative susceptibility of each formulation to aging-induced wear damage can be evaluated directly.

From Figure 9a–f it was demonstrated that, under 40 N, 1 Hz, and 100 m sliding, GF reinforcement reduces the baseline wear severity by decreasing the unaged wear depth from approximately 161 µm (neat PLA) to approximately 111 µm (10 wt.% GF) and approximately 94 µm (15 wt.% GF), while also reducing the wear track width from approximately 1789 µm to approximately 1620–1616 µm. Water aging increases wear damage in all systems; however, the aging duration associated with the maximum wear depth is formulation dependent. Neat PLA reaches its worst-case condition at 7 days, where the wear track expands to approximately 2201 µm in width and approximately 233 µm in depth, consistent with rapid matrix plasticization and enhanced deformation-driven penetration. In contrast, both GF-reinforced systems reach their maximum depths at 28 days, indicating a delayed but interface-controlled degradation response. The 10 wt.% GF composite increases to approximately 1923 µm in width and approximately 132 µm in depth, whereas the 15 wt.% GF composite exhibits the strongest deterioration in depth, increasing to approximately 157 µm despite a comparable width increase to approximately 1903 µm, which is consistent with progressive fiber–matrix debonding, microcracking, and debris-assisted three-body abrasion that promotes deeper penetration at higher fiber content.

The three-dimensional profilometric observations shown in Figure 8 and Figure 9 indicate that pressurized water aging leads to notable geometric changes in the wear tracks of both neat PLA and GF/PLA composites. The observed trends of increased depth and width of wear scar formation with increased durations of aging can be explained in light of existing knowledge on the degradation of such materials, where exposure to moisture results in a softened matrix with reduced load-carrying capability, thus facilitating wear scar formation of increased depth and width. Furthermore, the trends of wear scar formation in the GF/PLA composite materials, such as increased width of wear scar, asymmetrical wear scar profiles, and irregular wear scar formation, can be explained in light of existing knowledge on the degradation of such materials, where exposure to moisture results in weakening of the interfacial regions, exposure of fibers, and three-body abrasion caused by debris [46,47]. The trends of wear scar formation observed in this study can be said to align with the existing body of knowledge on the wear behavior of hydrothermally aged composite materials.

### 3.5. Microstructural Examination of Wear Damages

**Figure 10:**
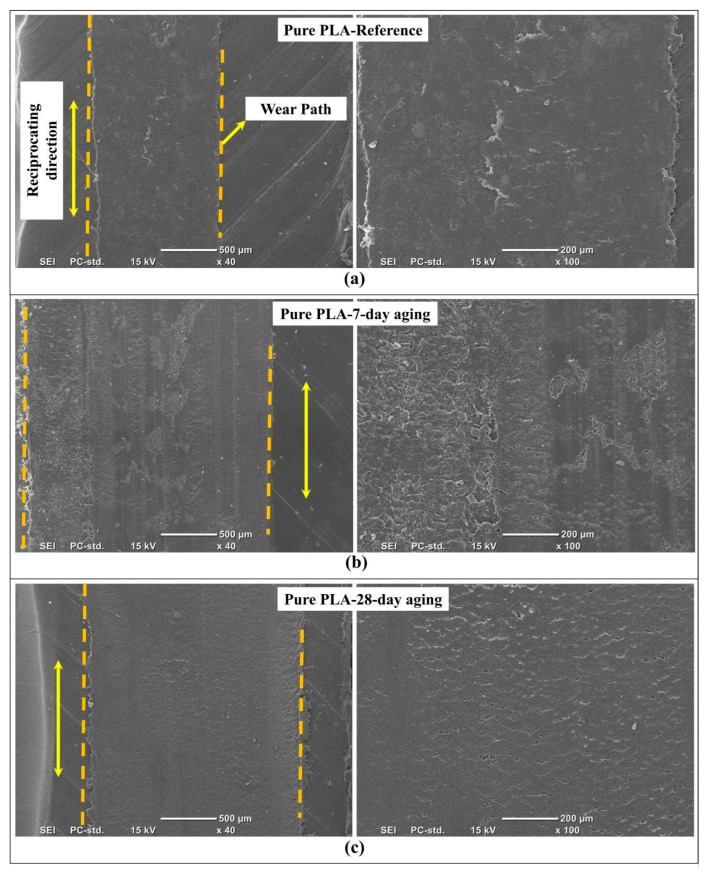
Wear damages of pure PLA; (**a**) reference-no aging; (**b**) 7-day aging; (**c**) 28-day aging.

Figure 10 provides representative SEM observations of the reciprocating wear tracks produced on pure PLA in the reference (no aging) state and after water aging for 7-day and 28-day. The low magnification views delineate the overall wear path and its lateral boundaries, while the higher magnification images resolve the dominant local damage modes inside the track, enabling a mechanism-level comparison of how water exposure modifies near-surface deformation, debris evolution, and tribo-layer stability during sliding.

In Figure 10a, the absence of aging in pure PLA shown in Figure 10a has a relatively even wear rate with less discontinuity over a wide wear track. The main wear appearance is of smearing and shallow micro-plowing scratches that are in line with the rubbing direction. This indicates that wear occurs in a manner that involves visco-plastic flow of the material. At a higher magnification, the wear track is relatively dense, but there are patches of film and microcrack details that are not so prominent. All these indicate that polymer flow due to visco-plastic material is involved in increased rubbing that may have led to a stable interface.

From Figure 10b, it is clear that the wear track with seven-day aging exhibits a significantly greater degree of surface damage than the reference sample. The low-magnification micrograph discloses an increased degree of irregularity within the wear track, as well as increased signs of surface disruption associated with debris, suggesting an increased ease of removal and dispersal of the broken-off polymer pieces during bidirectional rubbing. The high-magnification micrograph discloses extensive surface roughening, pronounced microcracking, as well as significant delamination at various locations, indicating an increased level of adhesive wear superimposed upon abrasion. This wear feature correlates with modifications to the near-surface mechanics induced by absorbed moisture, leading to decreased strength and increased compliance with attendant removal by subsequent rubbing-induced tearing, unstable third-body films, and third-body abrasion by debris pieces. Carrasco et al. [48] reported that hydrolytic aging of PLA predominantly affects the polymer chains located in the amorphous domains, resulting with a pronounced decrease in molecular weight and a consequent increase in matrix brittleness. This degradation creates a network of subsurface microcracks that, under the cyclic shear stress of reciprocating sliding, coalesce into the large-scale delamination observed.

The 28-day-aged pure PLA shown in the images of Figure 10c has a wear track that seems to be more uniform compared to the situation in the 7-day-aged PLA, despite the obvious signs of ongoing sliding-induced changes. Indeed, the appearance of the wear track under low magnification appears to be less dominated by delamination and to have a more homogeneous core, which may reflect the ongoing reduction in the wear process contributions from the large patch pull-off mechanisms that dominated the initial aging stage. Under higher magnification, the observed fine and directionally aligned groove structure and relatively compacted texture may also reflect the typical characteristics of an abrasion-controlled regime, which, under the action of micro-plowing and the relatively stabilized debris-compacted layer, seems to contrast the more homogeneous and microcrack-assisted spallation-dominated situation of the 7-day-aged material, where the dominant morphological changes seem to favor the transition to a more stationary sliding regime where the damage contribution to the wear process seems to be relatively more uniformly distributed along the contact patch. This observation is consistent with the results reported by Afrose et al. [49], who showed that extended exposure to water promotes the development of a weak and eroded surface layer on PLA. During sliding, this degraded layer is gradually eliminated through micro-plowing mechanisms, giving rise to the oriented groove patterns observed on wear tracks after long-term aging.

**Figure 11:**
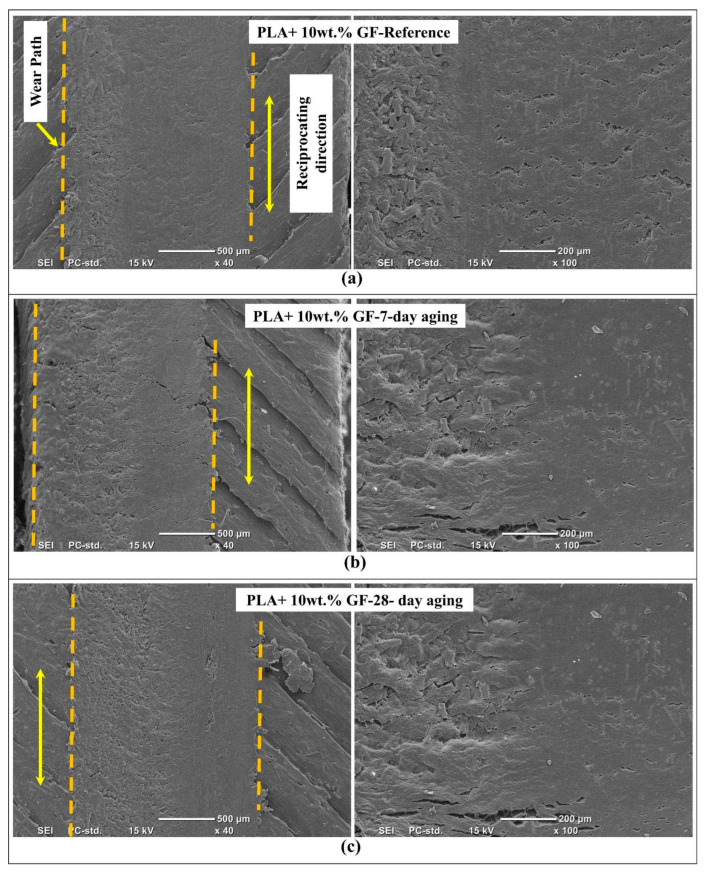
Wear damages of PLA + 10 wt.% GF composite; (**a**) reference-no aging; (**b**) 7-day aging; (**c**) 28-day aging.

Figure 11 compares the SEM morphologies of wear tracks formed on PLA reinforced with 10 wt.% glass fiber in the reference state and after water aging for 7-day and 28-day. The low magnification images define the wear path boundaries and the directionality of sliding, while the higher magnification views resolve the local damage mechanisms within the track, particularly matrix smearing, interfacial debonding, fiber exposure, and debris-driven abrasion. This figure therefore clarifies how introducing GF changes the dominant wear mode from purely matrix-controlled plastic flow toward a coupled matrix, fiber and interface response, and how water aging progressively alters that balance.

As can be seen from Figure 11a, the reference sample of PLA containing 10wt.% glass fiber (GF) has a wear track that is comparatively well-defined, reflecting a compact region in the wear track. The low magnification view also reveals that there is a wear track that is a continuous band and does not exhibit significant edge spallation. At higher magnification, the worn surface is characterized by smeared polymer patches and shallow grooves aligned with the reciprocating direction, together with discrete regions where short fiber ends or fiber imprints are visible. The presence of these imprints and limited pull-out cavities indicates that, in the unaged condition, the fiber–matrix interface largely remains intact, so fibers act primarily as barriers that resist penetration and stabilize the tribo-layer, rather than being released as abrasive third-body fragments.

Figure 11b shows that 7-day aging introduces a clear increase in surface disruption within the wear track. The low magnification image indicates a rougher track interior and more pronounced longitudinal shear bands, implying that the near-surface matrix becomes more compliant and prone to shear localization after water exposure. The higher magnification view reveals more frequent microcracks, torn polymer ligaments and small void like features associated with partial interfacial debonding or early-stage fiber pull-out. Compared with the reference, the worn surface appears less uniformly smeared and more fragmented, which suggests a transition from a predominantly adhesive smearing regime toward a mixed mechanism where locally detached polymer and partially loosened fibers promote intermittent third-body abrasion and micro cutting along the sliding direction.

In Figure 11c, it is observed that the PLA sample aged for 28 days with 10 wt. % glass fiber (GF) has been affected to the highest extent. This is consistent with it having recorded the highest value on depth response. In the low magnification image, it is observed that there is higher heterogeneity on the track, which is characterized by pronounced shearing or removal, and there is less clarity on boundaries. At higher magnification, the surface contains more extensive delamination patches, larger cavities consistent with fiber pull-out, and a higher density of grooves and scratches aligned with the reciprocating direction. These features indicate that long-term water aging substantially degrades the fiber–matrix interface, allowing fibers to debonding and fragment, and the released glass particles then act as hard third-body abrasives that intensify micro-plowing and deepen the wear track. Overall, Figure 10 demonstrates that glass fiber reinforcement improves the integrity of the unaged wear surface, but the tribological advantage progressively diminishes with aging as interfacial weakening activates debonding, pull-out, and abrasion-dominated wear.

**Figure 12:**
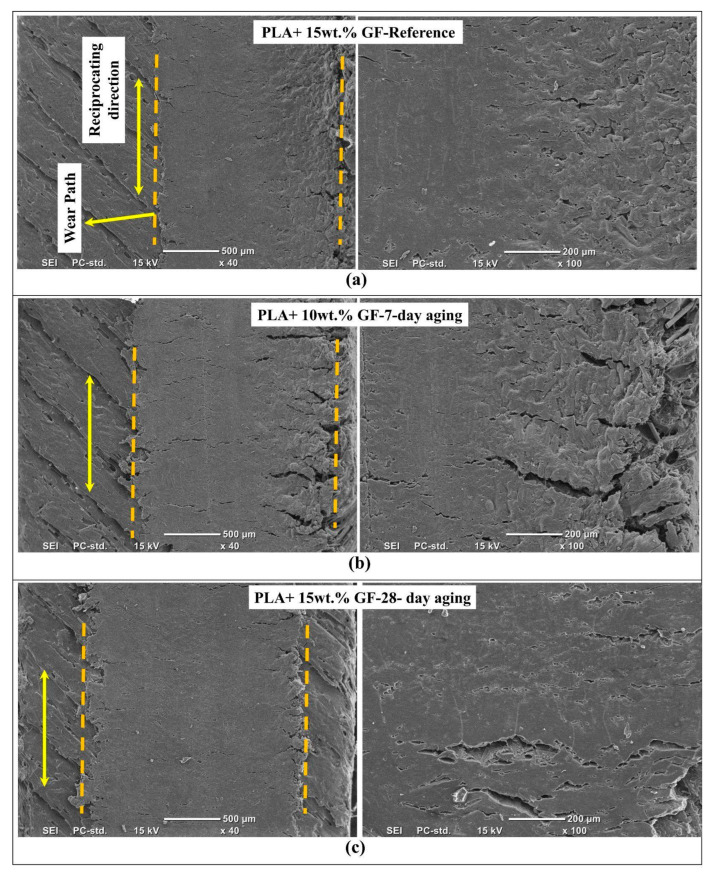
Wear damages of PLA + 15 wt.% GF composite; (**a**) reference-no aging; (**b**) 7-day aging; (**c**) 28-day aging.

Figure 12 presents SEM micrographs of reciprocating wear tracks formed on PLA reinforced with 15 wt.% glass fibers in the reference condition and after water aging for 7-day and 28-day. The low magnification images delineate the wear path boundaries and reveal the macroscopic continuity of the track, whereas the higher magnification views resolve the local micro mechanisms governing damage development, including matrix smearing, microcracking, interfacial debonding, and fiber-related damage features. Because the fiber fraction is higher than in the 10 wt.% system, the wear response is more strongly controlled by fiber crowding effects and localized stress concentrations at the fiber–matrix interface, thereby rendering the aging-related degradation of interfacial integrity particularly evident in the resulting wear track morphology.

Starting with Figure 12a, the reference PLA with 15 wt.% GF specimen displays a relatively compact wear track with a relatively homogeneous interior at a lower magnification. The wear track retains distinguishable boundaries without signs of large-scale spalling along these boundaries, which indicates a capacity of the modified surface to withstand the load without major displacement of materials. Higher magnification of the wear track reveals a surface with shallowed grooves oriented in the direction of motion as well as a smeared polymer with microcracks. These features confirm a mixed wear mechanism in which the visco-plastic deformation of the softened polymer is restricted by the high fraction of fibers, thus favoring microcracking within regions of high stochastic stress. The paucity of signs of large pull-out voids in the virgin structure indicates a strong enough bond between the interfacial zones to resist large-scale de or rebonding of the fibers within the wear track.

Figure 12b corresponds to the 7-day-aged condition of the PLA + 15 wt.% GF composite. Compared with the reference, the low magnification image shows a more disrupted track edge region and a more heterogeneous interior, indicating that aging accelerates damage localization and promotes intermittent material removal. The higher magnification view reveals a clear increase in microcrack density and crack opening, together with torn polymer ligaments and zones where the surface appears fractured and fragmented rather than smoothly smeared. This morphology is consistent with moisture-induced weakening of the matrix and, critically, partial loss of interfacial cohesion, which facilitates debonding at the fiber–matrix boundary and promotes crack propagation along interface-rich regions. The result is a more brittle, crack-assisted wear process in which local delamination events and debris generation become more frequent, increasing the likelihood of third-body abrasion during reciprocating sliding. The panel header in the image appears to contain a typographical inconsistency, and it should be aligned with the caption to indicate the PLA + 15 wt.% GF, 7-day aging condition.

In Figure 12c, the 28-day-aged PLA + 15 wt.% GF depicts an ongoing process in the wear mechanism. By observing the low-magnification view, there is an onset of a more uniformly sheared path for the wear process in contrast to the 7-day-aged image. At higher magnification, the surface shows extended, directionally aligned grooves and elongated cavities or gaps that are consistent with advanced interfacial debonding and partial fiber pull-out or fiber imprinting. The presence of these elongated defects, together with the less continuous smeared film compared with the reference, indicates that long-term aging promotes progressive interface deterioration, enabling fibers to loosen and act as hard asperities or debris sources. Under reciprocating motion, such released or partially exposed fibers can intensify micro-plowing and micro cutting, thereby deepening the wear track and increasing surface roughness inside the path. Overall, Figure 12 confirms that while 15 wt.% GF reinforcements can stabilize the unaged wear track, water aging shifts the dominant damage mode toward interface-controlled cracking and debonding, and this shift becomes increasingly pronounced as the exposure duration increases. In the hydrothermal aging of glass fiber/epoxy composites conducted by Yao et al. [50], one can observe a clear similarity to the problem being discussed. The scanning electron microscopy results showed the same debonding along the interfacial boundaries between the fibers and matrices, which the authors believed to be due to the hydrolysis of the silane coupling agent and the infiltration of water molecules, reducing the strength of the adhesion. Once debonded, these glass fibers and fragments become potent third-body abrasives. The severe plowing and deep, sharp grooves in the aged composites are hallmarks of this mechanism.

**Figure 13:**
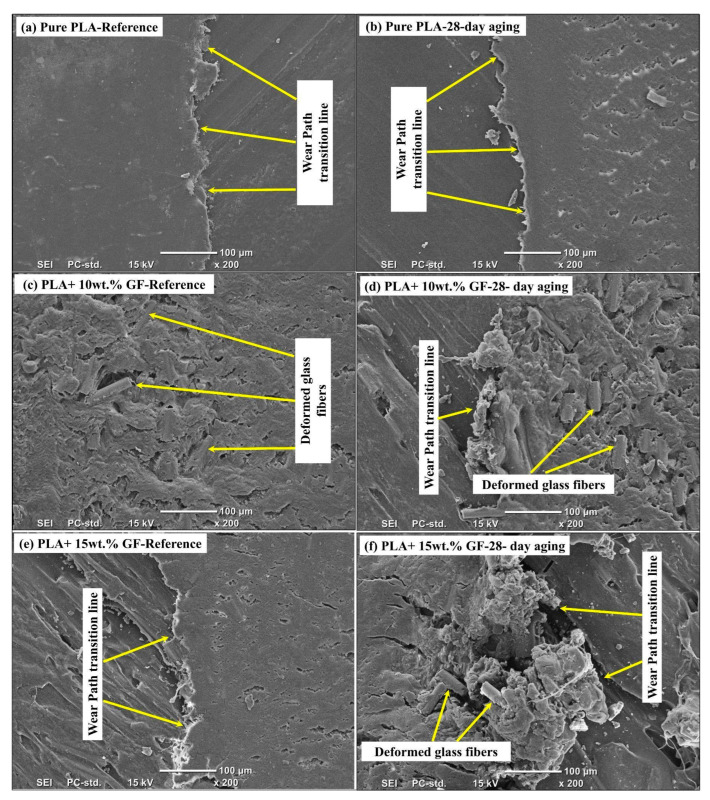
Wear damages of transition zones; (**a**) pure PLA, reference-no aging; (**b**) pure PLA, 28-day aging; (**c**) PLA + 10 wt.% GF, reference-no aging; (**d**) PLA + 10 wt.% GF, 28-day aging; (**e**) PLA + 15 wt.% GF, reference-no aging; (**f**) PLA + 15 wt.% GF, 28-day aging.

Figure 13 illustrates the wear track transition zone that has been defined as the boundary of the worn region and the less affected area that surrounds it. This interface is mechanistically important because it concentrates shear stresses, governs the stability of the tribo-layer, and is often the initiation site for edge delamination, crack growth, and debris release. The SEM micrographs therefore complement the width and depth data by revealing how aging and glass fiber addition change the local failure mode at the track margin, shifting the balance between ductile smearing, interfacial separation, and abrasion by third-body particles.

In Figure 13a, the reference pure PLA shows a relatively well-defined wear path transition line with limited edge fragmentation. The boundary is shown to be in the form of a ridge that delineates a smooth worn zone from the surrounding material and suggests that mainly matrix shearing is occurring. In Figure 13b, after 28-day aging, the same transition line becomes more irregular and locally undercut, with a rougher adjacent surface populated by small cavities and torn features. This change indicates that long-term water exposure reduces the cohesive integrity of the near-surface PLA, promoting localized edge chipping and discontinuous delamination at the boundary, which would facilitate intermittent debris generation and a less stable tribo-layer compared with the reference condition.

Figure 13c shows the transition region for the unaged PLA + 10 wt.% GF composite. The boundary is less purely ridge-like than in pure PLA and is accompanied by distinct deformed glass-fiber-related features within and near the transition zone. The local texture is more heterogeneous, consistent with load sharing between matrix and fibers and with shear localization around fiber-rich regions. In Figure 13d, the 28-day-aged PLA + 10 wt.% GF specimen exhibits a markedly more disrupted transition line, together with more prominent clusters of deformed, partially exposed, or displaced glass fibers. The morphology suggests that aging weakens the fiber matrix interface, enabling fibers to debonding and protrude into the contact, while simultaneously producing hard fragments that intensify micro-plowing at the wear track edge. As a result, the transition zone evolves from a relatively coherent boundary in the reference state into a mechanically rough interface dominated by mixed adhesive and abrasive mechanisms.

In Figure 13e, there is an observable transition line in the unaged PLA reinforced by 15wt.% GF, and there is also increased edge cracking and discontinuity compared to the reference sample of 10wt.% GF. This is consistent with higher fiber crowding and increased stress concentrations at the boundary, where matrix ligaments between adjacent fibers are thinner and more prone to crack-assisted separation under cyclic shear. In Figure 13f, after 28-day aging, the transition zone becomes the most severely disrupted among the six panels. Large agglomerated debris masses and strongly deformed glass fiber bundles are visible adjacent to the boundary, indicating extensive interfacial debonding, fiber pull-out or fracture, and compaction of mixed polymer fiber debris into a mechanically active third-body layer. This morphology implies that the contact in the aged 15 wt.% GF composite is strongly influenced by abrasive action of glass-rich debris and by repeated edge delamination events, which are expected to accelerate track deepening and destabilize friction compared with both the unaged 15 wt.% GF specimen and the aged 10 wt.% GF composite. Across all materials, two consistent trends emerge from Figure 13. First, water aging transforms the wear track boundary from a relatively continuous transition line into a more irregular, locally delaminated interface, indicating reduced near-surface cohesion and less stable tribo-layer formation. Second, introducing glass fibers moves the controlling mechanism at the transition zone from matrix-dominated smearing toward interface-controlled damage. The effect becomes stronger at 15 wt.% GF, where fiber crowding and aging-induced interfacial weakening promote severe debris accumulation and fiber-dominated third-body abrasion at the boundary. Sapuan et al. [51], in the analysis of composite failures, highlighted that moisture absorption reduces the matrix’s fracture toughness and promotes crack initiation at stress concentrators (such as fiber ends or voids).

### 3.6. DSC Analyses

**Figure 14:**
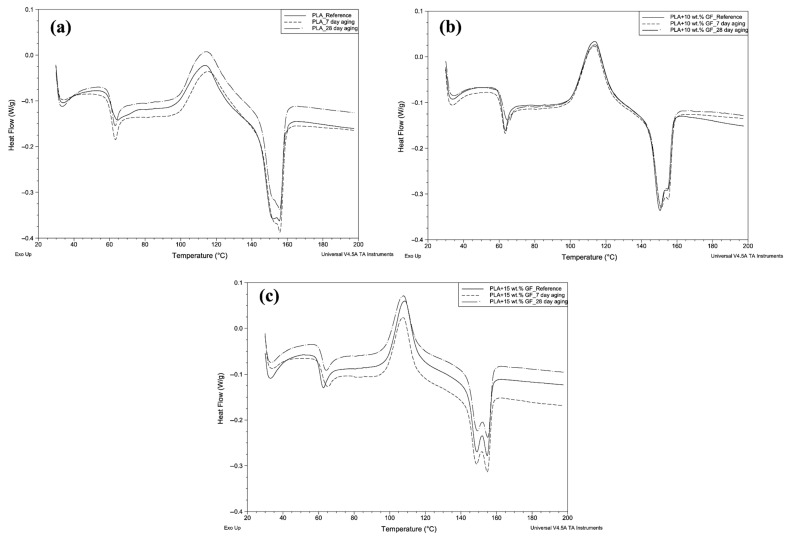
DSC thermograms of samples: (**a**) water-aged pure PLA; (**b**) water-aged PLA + 10 wt.% GF samples; (**c**) water-aged PLA + 15 wt.% GF samples.

The variations in the thermal properties of 3D-printed samples produced by 3D printing using three different filament types due to water aging for varying periods of time were investigated by DSC analysis. Although the water aging process was applied for 0 (reference), 7-day, 14-day and 28-day, DSC analyses were only applied to samples aged for 0, 7-day and 28-day. The methodology was designed to elucidate the progressive impact of water aging on the thermal response of the material, capturing changes occurring at the early, intermediate, and advanced stages of exposure. In this context, DSC thermograms showing the result of three different water aging processes applied to the pure PLA sample are presented comparatively in Figure 14a–c and Table 3.

It is seen that in Figure 14a, all DSC curves of pure PLA, T_g_, T_cc_ and T_m_ peaks, which are considered to be characteristic for PLA, can be clearly observed. However, the DSC curves given in Figure 14a and Table 3 also show that some changes have occurred in the thermal behavior of water-aged pure PLA samples. While no important change is seen in Tg depending on the water aging time, it is observed that T_cc_ values increase slightly with aging. This increase can be explained by the shortening of PLA chains as a result of hydrolytic degradation and thus the increase in the number of chain ends. It can be said that the shortened PLA chains need more free volume and energy to be able to cold crystallize, that is, to start to become more regular, and therefore the T_cc_ value shifts to higher temperatures. At the same time, the increase observed in ΔH_cc_ value at 28 days of water aging can be interpreted as the water aging process applied for a relatively longer period of time facilitating the cold crystallization of the polymer chains by regularizing them, i.e., increasing their crystallization tendency. In addition to all these results, it can be concluded from Figure 14 that Tm was observed at similar values in all three samples, and small changes occurred in ΔH_m_ values due to water aging, but these changes were not at a level that would significantly affect the crystal structure [52,53,54]. In general, T_g_ and T_m_ of PLA did not change under water aging conditions, but water aging has the potential to cause structural changes that may increase the crystallization tendency. The thermal transition behaviors of PLA composites containing 10 wt.% GF at 0-day, 7-day and 28-day water aging conditions are given in Figure 14b and Table 3.

When Figure 14b and Table 3 are analyzed, it is seen that the T_g_ value does not change in all three sample types. This can be interpreted as GF addition to pure PLA does not have a significant effect on the chain mobility of PLA. The result that no change was observed in the chain mobility of PLA despite the addition of 10 wt.% GF suggests that the added glass fibers are micron-scale particles with a diameter in the range of 10–13 μm and particle size in the range of 50–200 μm, and due to their relatively low size, they do not form a strong interaction at this rate and size to restrict PLA chain mobility [55]. However, as can be seen from Figure 14b and Table 3, T_cc_ values do not show a significant change depending on the water aging time. When this result is compared with the results of pure PLA given in Figure 14a, it can be interpreted that the cold crystallization behavior of PLA chains becomes more stable with the addition of GF, which is not affected by water aging. Although small changes were observed in ΔH_cc_ and ΔH_m_ values depending on the water aging time, these changes cannot be said to be significant changes in the general structure of the crystal phase. In fact, Tm also exhibited very close values in all sample types, which once again showed that water aging had no significant effect on the crystal structure. In conclusion, it has been observed that adding 10 wt.% GF was not found to induce a change in the thermal properties of PLA matrix composites; however, it limits the change in thermal properties by water aging to a certain extent. The thermal transition behaviors of PLA composites containing 15 wt.% GF at 0-day, 7-day and 28-day water aging conditions are given in Figure 14c and Table 3.

From the analysis of Figure 14c and Table 3, it can be observed that the Tg values are generally constant during different aging durations. However, a slight increase in Tg (1.5 °C) is observed for the PLA composite containing 15 wt.% GF after water aging. Although the increased fiber content may slightly restrict the segmental mobility of the PLA chains, this phenomenon did not fully explain the observed change, as no such increase was observed before aging. Therefore, it is possible that the water aging process promotes interfacial interactions between the PLA matrix and glass fibers, such as the formation of hydrogen bonds, which may further limit chain mobility and contribute to the increase in T_g_. Moreover, as evident from Figure 14c and Table 3, the cold crystallization temperature (T_cc_) remains largely unchanged despite increasing water aging duration; the values of ΔH_cc_ decrease significantly with the increase in water aging time. Nevertheless, this decrease in values of ΔH_cc_ does not show a rise with increasing aging time. It can be assumed that this decrease in values of ΔH_cc_ could be attributed to a diminished capacity of the PLA chains caused by hydrolytic degradation because of aging. The effect of 15 wt.% GFs might be related to restricting the crystallization of PLA chains due to regularizing [56].

### 3.7. Factorial Analysis

The factorial analysis can be defined as a statistical approach that helps to test more than one factor for its effect on a variable by considering all levels of variables. This approach to data collection is more effective compared to the traditional one-factor-at-a-time approach to data collection in experimental studies. This is because it saves experimental costs and time by considering all levels of variables. This approach is more effective due to its capacity to increase statistical credibility of experimental findings by providing knowledge on complex interdependencies between different variables using a unified approach. This paper uses factorial analysis to determine the effects of several critical parameters such as glass fiber weight ratio (0%, 10%, and 15%), test frequency (0.5 Hz and 1 Hz), and aging duration with pressurized water (0 to 28-day) on wear track properties, as well as coefficient of friction (COF), by considering their contribution effects.

As per the result obtained from the General Factorial Regression test, it can be interpreted that the overall fit of the model is good, as it has a high value of explanation with an R-squared of 97.43% and an adjusted R-squared of 96.34%. This means that more than 97% of the total variability of the coefficient of friction (COF) is explained by the variables of interest, which are factors of sample type, aging time, and test frequency. As can be seen in Table 4, the main contributing factors to the COF values are outlined alongside their contribution percentages. The variance analysis has revealed the parameter that has the highest contribution to the COF values. This is the immersion time in water. This can be justified by the hydrolytic degradation process as outlined in this paper. Immersion in pressurized water will result in plasticization of the material through water uptake. sample type (PLA vs. GF-PLA) is the second most significant variable in the model. The statistical results confirm that glass fiber addition increases wear resistance and reduces friction variability compared to PLA. However, the interaction of GF-PLA on the degradation of the interface in the water aging test increases the importance of the relationship between sample type (PLA vs. GF-PLA) and “Water Aging Time”. Wear test frequency (Hz) is an important factor in COF, but its contribution ratio is smaller than other two major variables, which shows that the properties of the material (aging and reinforcement ratio) have more importance than other factors related to conducting the test (speed or frequency). Interaction: The sample × water immersion duration interaction is very interesting.

The high significance and contribution rate of three-way interactions in the analysis results prove that the effects of the controlled parameters on the friction coefficient are not independent of each other, but rather that the variables act with a collective synergy. High interaction is that the hydrolytic degradation caused by pressurized water triggers both the plasticization of the polymer matrix and the weakening of the glass fiber–matrix interface, thus altering the wear resistance of the material through a complex and variable mechanism depending on the sample type and test frequency. The high value of the three-way interaction in the analysis results proves that the combined effect of sample type, aging time, and test frequency on the friction coefficient exhibits a much more complex and interdependent characteristic than the individual contributions of these factors. The main reason for this is that the microstructural degradations caused by water in the polymer matrix and fiber interface, combined with dynamic loading under different test frequencies, trigger a unique and nonlinear wear mechanism for each material group.

**Figure 15:**
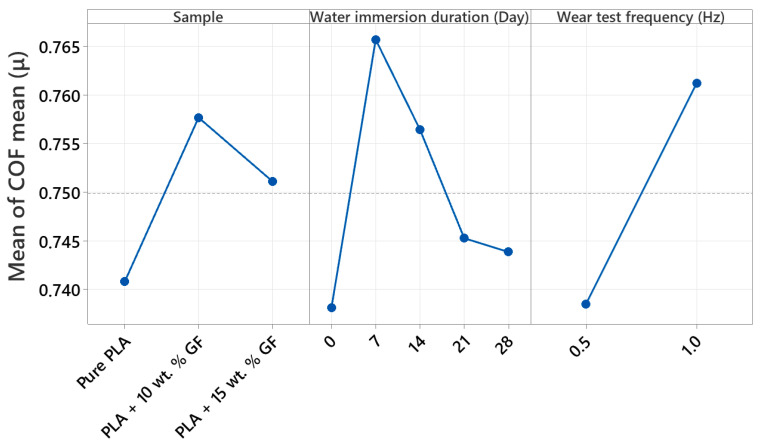
Main effects plot for the mean friction coefficient (COF) illustrating the individual impact of sample type, water immersion duration, and sliding frequency.

Figure 15 illustrates the individual effects of control parameters (sample type, water aging time, and test frequency) on the mean coefficient of friction (COF) through a “Main Effects Plot”. The steepness of the slope between the data points in the graph directly represents the intensity of the effect of the respective parameter on COF. Analysis reveals that water aging time has the steepest slope on the graph and leads to the most significant change in COF values. This confirms that plasticization and micro-scale structural distortions caused by pressurized water in the PLA matrix dominate the tribological response. The significance of a sample-type analysis lies in its ability to articulate well the difference in friction force due to reinforcement by glass fibers compared to PLA alone, wherein the reinforcement components act as load carriers on the wear surface. By doing this, it changes or affects the mechanism of friction force. Secondly, the variable of test frequency has less significance as a main factor in influencing the coefficient of friction (COF), and it plays a supplementary role in the interaction processes explained in the preceding section. Figure 15 above is crucial in showing what factor takes priority in regulating the coefficient of friction.

From Table 5, it can be seen that the major source of variation in the width of the wear track was the immersion time in water, accounting for 76.02% of the variation. This value underlines the high vulnerability of the material’s tribological properties in terms of environmental conditions, particularly pressurized water and immersion time. For both PLA and GF-PLA samples, the main mechanism in defining the width of wear was identified as being due to chemical dissolution/matrix softening due to water. The contribution of the sample type (sample) and the test frequency (Hz) was 5.76% and 4.14%, respectively. These small percentages imply that the material properties in terms of microstructure, affected by water immersion, and testing speed have negligible effects on the width of the wear tracks. Beyond the individual parameters, the sample × water immersion duration interaction has a contribution share of 5.09%. This percentage shows that the response of the sample type (pure or reinforced) to water differs; this numerically confirms that the water-based wear track widening effect progresses with different intensities (synergistic effect) on GF-PLA and pure PLA. The triple interaction (sample × water × frequency) being 1.40% and the model error margin being only 2.57% demonstrates that the experimental design is extremely precise and that the obtained width data are not coincidental but a direct result of the complex interaction of these three parameters.

**Figure 16:**
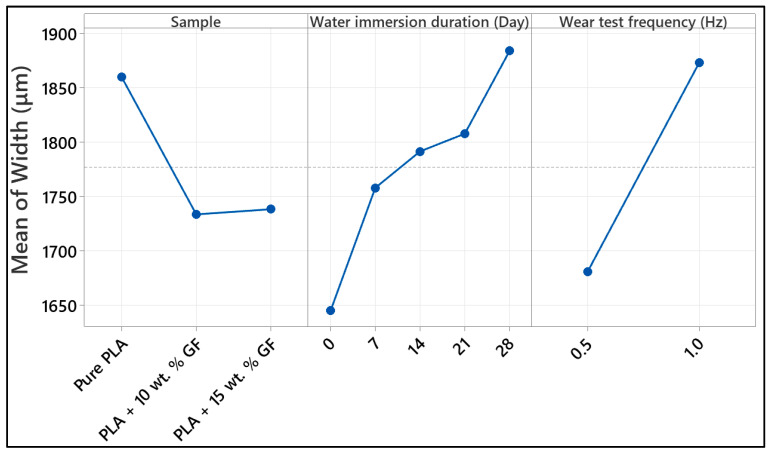
Variation in mean wear track width as a function of experimental parameters: A main effects analysis based on General Factorial Regression for 3D-printed PLA and GF-PLA composites.

The main effects graph presented in Figure 16 clearly shows that the most dramatic change in wear track width occurs with “Water Immersion Duration”. The steep slope of the aging time curve shown in the graph above supports the high contribution rate of 76.02% shown in Table 5. The linear increase in the width of the wear track from 0 to 28-day can be attributed solely to the softening effects of the pressurized water on the polylactic acid (PLA) matrix. In determining the differences in variation based on the type of samples used, it can be seen that the glass fiber (GF) additives resist the width of the wear track, but this tends to weaken with time when exposed to water. In the case of the test frequency (Hz) factor, the slope follows a more horizontal course, proving that the frequency variation does not play as dominant a role in wear track width as the microstructural state (aging level) of the material. In summary, this graph scientifically demonstrates that wear track width is extremely sensitive to material intrinsic strength loss (hydrolytic degradation), but less affected by the mechanical speed of the test.

It is seen in Table 6 that the most dominant factor on wear depth is “Water Immersion Duration”. The contribution rate of this parameter is the highest, similar to (and generally higher than) the width data in Table 5. This demonstrates that the hydrolytic degradation caused by pressurized water not only creates a surface effect but also causes deep softening (plasticization) of the polymeric structure, leading to easier penetration of the abrasive tip into the material. The high contribution rate of water immersion duration shows that the structural integrity of the material weakens in direct proportion to the time spent underwater. In pure PLA and GF-PLA samples, micro-voids and bond breaks resulting from the diffusion of water within the matrix are the primary cause of the increase in wear depth. The fact that the sample type’s contribution ratio remains secondary to the aging time, while limiting the vertical wear depth of the glass fiber reinforcement to some extent, indicates that the softening of the matrix with water dominates this protective effect. Similarly, the low contribution of frequency (Hz) documents that the vertical material loss (depth) is related to the material’s current hardness/softness state rather than the test speed.

**Figure 17:**
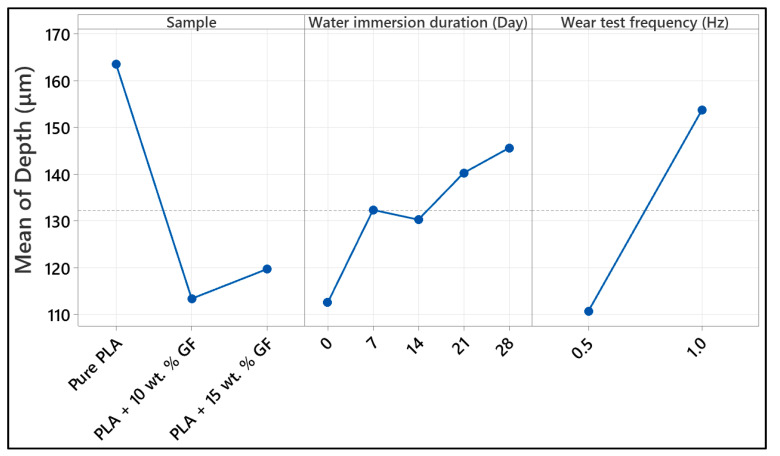
Main effects plot for the mean wear depth, illustrating the comparative influence of material composition, aging duration, and testing frequency based on factorial regression analysis.

The main effects graph presented in Figure 17 visually demonstrates that sample type and test frequency (Hz) are the most decisive factors influencing wear depth. According to the variance analysis results in Table 6, sample type is the primary parameter dominating vertical wear with a contribution rate of 34.45% and test frequency with 31.98%. Examining the sample type curve in the graph, it is seen that glass fiber reinforcement (GF) significantly limits the wear depth compared to pure PLA; however, this resistance performance changes over time due to the effect of water aging. The steep slope created by the increase in test frequency on the wear depth shows that the dynamic loading rate directly affects the vertical penetration resistance of the material. Although the direct (linear) effect of water aging on depth has a lower percentage compared to the width data, the softening of the material due to hydrolytic degradation contributes to the deepening of the wear profile.

## 4. Conclusions

Pressurized water aging at 10 bar markedly couples moisture uptake with the reciprocating tribological performance of FDM-printed neat PLA and glass-fiber-reinforced PLA (10 wt.% and 15 wt.% GF). Water absorption followed a Fickian trend and the primary effect of GF was to increase moisture capacity rather than to alter diffusion kinetics, as the saturation mass increased from 0.0845 g (PLA) to 0.1625 g (10 wt.% GF) and 0.295 g (15 wt.% GF), while diffusion coefficients remained narrowly clustered around 4.31 × 10^−12^ to 4.52 × 10^−12^ m^2^/s. Under 40 N and 100 m sliding, aging generally increased COF and wear in a frequency and reinforcement-dependent manner, including a non-monotonic response for 15 wt.% GF where the 21-day condition produced the lowest sustained COF at 0.5 Hz. GF reduced unaged wear depth from about 125 µm (PLA) to about 85 to 96 µm, yet prolonged immersion diminished this advantage and, at 0.5 Hz and 28 days, reinforced samples reached about 1830 to 1860 µm width and about 129 to 132 µm depth, while neat PLA exhibited a smaller scar (about 1625 µm width and 107 µm depth). At 1 Hz, wear depth became more severe, with neat PLA peaking at about 235 to 240 µm after 7-day, whereas 15 wt.% GF reached about 160 µm after 28 days. Factorial analysis further confirms that immersion duration dominates wear scar width (76.02% contribution), whereas wear depth is governed mainly by sample type (34.45%) and test frequency (31.98%), highlighting that pressurized moisture exposure can outweigh compositional benefits during long-term reciprocating service.

## Figures and Tables

**Table 1:** Wear test parameters.

Composite Type	Pure PLA PLA + %10 wt. GF PLA + %15 wt. GF
Load	40 N
Reciprocating Wear Test Track	100 m
Wear test frequency	0.5 Hz 1 Hz
Water aging duration	Reference (no aging) 7-day 14-day 21-day 28-day

**Table 2:** Diffusion parameters of PLA and its composites at 10 bar pressure.

Sample Type	M∞ (g) (Balance Mass)	Initial Slope (k) (g·s^−0.5^)	Diffusion Coefficient (D) (m^2^/s)
PLA	0.0845	9.90 × 10^−5^	4.31 × 10^−12^
PLA + wt.% 10 GF	0.1625	1.91 × 10^−4^	4.38 × 10^−12^
PLA + wt.% 15 GF	0.2950	3.54 × 10^−4^	4.52 × 10^−12^

**Table 3:** Thermal transition temperatures and enthalpy values of PLA-based samples.

Composite Type Thermal Properties	Pure PLA			PLA + 10 wt.% GF			PLA + 15 wt.% GF		
	Reference	7-Day Aging	28-Day Aging	Reference	7-Day Aging	28-Day Aging	Reference	7-Day Aging	28-Day Aging
T_g_ (°C)	61.17	61.66	61.63	62.12	62.13	62.51	61.01	62.21	62.37
T_cc (onset)_ (°C)	97.38	99.57	99.72	100.96	100.71	100.37	98.64	98.25	97.75
T_m (peak)_ (°C)	155.48	155.72	155.73	150.49	151.22	150.99	154.75	154.96	155.06
ΔH_cc_ (J/g)	22.61	22.01	25.67	24.21	22.96	24.25	21.21	16.54	17.92
ΔH_m_ (J/g)	28.56	28.54	27.52	23.06	24.56	24.34	21.46	20.36	20.02

**Table 4:** General Factorial Regression analysis results for the mean COF, illustrating the statistical significance and contribution percentages of sample type, water immersion duration, and wear test frequency.

Source	DF	Seq SS	Contribution	Adj SS	Adj MS	F-Value	*p*-Value
Model	30	0.10109	95.93%	0.10109	0.00337	22.8	0
Blocks	1	0.000058	0.06%	0.000058	0.000058	0.39	0.536
Linear	7	0.016485	15.64%	0.016485	0.002355	15.94	0
Sample	2	0.002886	2.74%	0.002886	0.001443	9.77	0.001
Water immersion duration (Day)	4	0.005869	5.57%	0.005869	0.001467	9.93	0
Wear test frequency (Hz)	1	0.007729	7.34%	0.007729	0.007729	52.3	0
2-Way Interactions	14	0.060781	57.68%	0.060781	0.004342	29.38	0
Sample × Water immersion duration (Day)	8	0.034155	32.41%	0.034155	0.004269	28.89	0
Sample × Wear test frequency (Hz)	2	0.023895	22.68%	0.023895	0.011948	80.85	0
Water immersion duration (Day) × Wear test frequency (Hz)	4	0.002731	2.59%	0.002731	0.000683	4.62	0.005
3-Way Interactions	8	0.023766	22.55%	0.023766	0.002971	20.1	0
Sample × Water immersion duration (Day) × Wear test frequency (Hz)	8	0.023766	22.55%	0.023766	0.002971	20.1	0
Error	29	0.004285	4.07%	0.004285	0.000148		
Total	59	0.105376	100.00%				

**Table 5:** General Factorial Regression results for wear track width including statistical significance levels and percentage contributions of factors.

Source	DF	Seq SS	Contribution	Adj SS	Adj MS	F-Value	*p*-Value
Model	30	1,809,747	98.52%	1,809,747	60,325	64.29	0
Blocks	1	7	0.00%	7	7	0.01	0.933
Linear	7	1,128,771	61.45%	1,128,771	161,253	171.85	0
Sample	2	205,653	11.20%	205,653	102,826	109.58	0
Water immersion duration (Day)	4	366,312	19.94%	366,312	91,578	97.59	0
Wear test frequency (Hz)	1	556,807	30.31%	556,807	556,807	593.39	0
2-Way Interactions	14	532,859	29.01%	532,859	38,061	40.56	0
Sample × Water immersion duration (Day)	8	135,462	7.37%	135,462	16,933	18.05	0
Sample × Wear test frequency (Hz)	2	314,311	17.11%	314,311	157,155	167.48	0
Water immersion duration (Day) × Wear test frequency (Hz)	4	83,087	4.52%	83,087	20,772	22.14	0
3-Way Interactions	8	148,110	8.06%	148,110	18,514	19.73	0
Sample × Water immersion duration (Day) × Wear test frequency (Hz)	8	148,110	8.06%	148,110	18,514	19.73	0
Error	29	27,212	1.48%	27,212	938		
Total	59	1,836,959	100.00%				

**Table 6:** General Factorial Regression results for wear depth, illustrating the statistical significance and percentage contributions of sample type, water immersion duration, and wear test frequency.

Source	DF	Seq SS	Contribution	Adj SS	Adj MS	F-Value	*p*-Value
Model	30	86,416.5	99.81%	86,416.5	2880.6	495.81	0
Blocks	1	2	0.00%	2	2	0.35	0.56
Linear	7	65,147.2	75.24%	65,147.2	9306.7	1601.91	0
Sample	2	29,825.2	34.45%	29,825.2	14,912.6	2566.82	0
Water immersion duration (Day)	4	7629.9	8.81%	7629.9	1907.5	328.32	0
Wear test frequency (Hz)	1	27,692	31.98%	27,692	27,692	4766.46	0
2-Way Interactions	14	17,939.8	20.72%	17,939.8	1281.4	220.56	0
Sample × Water immersion duration (Day)	8	3453.6	3.99%	3453.6	431.7	74.31	0
Sample × Wear test frequency (Hz)	2	12,488.6	14.42%	12,488.6	6244.3	1074.8	0
Water immersion duration (Day) × Wear test frequency (Hz)	4	1997.6	2.31%	1997.6	499.4	85.96	0
3-Way Interactions	8	3327.5	3.84%	3327.5	415.9	71.59	0
Sample × Water immersion duration (Day) × Wear test frequency (Hz)	8	3327.5	3.84%	3327.5	415.9	71.59	0
Error	29	168.5	0.19%	168.5	5.8		
Total	59	86,585	100.00%				

## Data Availability

The datasets presented in this article are not readily available because the data are part of an ongoing study. Requests to access the datasets should be directed to corresponding author.

## Abbreviations

The following abbreviations are used in this manuscript:

PLA	Polylactic acid
GF	Glass fiber
FDM	Fused deposition modeling
Tg	Glass transition temperature
Tcc	Cold crystallization temperature
Tm	Melting temperature
ΔHcc	Cold crystallization enthalpy
ΔHm	Melting enthalpy
DSC	Differential scanning calorimetry
COF	Coefficient of friction
SEM	Scanning electron microscope
ANOVA	Analysis of variance
